# Supplementary material for: Mental health of Urban Mothers (MUM) study: a multicentre randomised controlled trial, study protocol
Source: BMJ Open. 2020 Nov 27;10(11):e041133. doi: 10.1136/bmjopen-2020-041133 (PMC7703424; doi:10.1136/bmjopen-2020-041133)
Supplement: Author's manuscript [file bmjopen-2020-041133.draft_revisions.pdf]

BMJ Open is committed to open peer review. As part of this commitment we make the peer review history of every article we publish publicly available.

When an article is published we post the peer reviewers' comments and the authors' responses online. We also post the versions of the paper that were used during peer review. These are the versions that the peer review comments apply to.

The versions of the paper that follow are the versions that were submitted during the peer review process. They are not the versions of record or the final published versions. They should not be cited or distributed as the published version of this manuscript.

BMJ Open is an open access journal and the full, final, typeset and author-corrected version of record of the manuscript is available on our site with no access controls, subscription charges or pay-per-view fees (http://bmjopen.bmj.com).

If you have any questions on BMJ Open's open peer review process please email info.bmjopen@bmj.com

BMJ Open

Mental Health of Urban Mothers (MUM) Study: A multi-center randomized controlled trial

Journal:	*BMJ Open*
Manuscript ID	bmjopen-2020-041133
Article Type:	Protocol
Date Submitted by the Author:	01-Jun-2020

Complete List of Authors:	Schwank, Simone Eliane; Karolinska Institute, CLINTEC; Columbia University, Center for Psychoanalytic Training and Research<br>Chung, Ho-Fung; The University of Hong Kong, Psychiatry<br>Hsu, Mandy; Johns Hopkins University, Psychological and Brain Sciences<br>Fu, Shih-Chien; National Taipei University of Education, Counseling Psychology<br>Du, Li; Tongji University, Department of Research and Education<br>Zhu, Liping; Tongji University, Department of Research and Education<br>Huang, Hsuan-Ying; The Chinese University Hong Kong, Anthropology<br>Andersson, Ewa; Karolinska Institutet, Department of Women´s and Children´s Health<br>Acharya, G; Karolinska Institutet,

Keywords:	Depression & mood disorders < PSYCHIATRY, Anxiety disorders < PSYCHIATRY, PERINATOLOGY, INFECTIOUS DISEASES, Reproductive medicine < GYNAECOLOGY

SCHOLARONE™ Manuscripts

I, the Submitting Author has the right to grant and does grant on behalf of all authors of the Work (as defined in the below author licence), an exclusive licence and/or a non-exclusive licence for contributions from authors who are: i) UK Crown employees; ii) where BMJ has agreed a CC-BY licence shall apply, and/or iii) in accordance with the terms applicable for US Federal Government officers or employees acting as part of their official duties; on a worldwide, perpetual, irrevocable, royalty-free basis to BMJ Publishing Group Ltd ("BMJ") its licensees and where the relevant Journal is co-owned by BMJ to the co-owners of the Journal, to publish the Work in this journal and any other BMJ products and to exploit all rights, as set out in our licence.

The Submitting Author accepts and understands that any supply made under these terms is made by BMJ to the Submitting Author unless you are acting as an employee on behalf of your employer or a postgraduate student of an affiliated institution which is paying any applicable article publishing charge ("APC") for Open Access articles. Where the Submitting Author wishes to make the Work available on an Open Access basis (and intends to pay the relevant APC), the terms of reuse of such Open Access shall be governed by a Creative Commons licence – details of these licences and which Creative Commons licence will apply to this Work are set out in our licence referred to above.

Other than as permitted in any relevant BMJ Author's Self Archiving Policies, I confirm this Work has not been accepted for publication elsewhere, is not being considered for publication elsewhere and does not duplicate material already published. I confirm all authors consent to publication of this Work and authorise the granting of this licence.

For peer review only

Mental Health of Urban Mothers (MUM) Study: A multi-center randomized controlled trial

Simone Schwank*, Karolinska Institutet, Department of Clinical Science Intervention and Technology (CLINTEC), Division of Obstetrics and Gynecology, Stockholm, Sweden

Chung, Ho-Fung, The University of Hong Kong, Department of Psychiatry, Hong Kong

Mandy Hsu, Johns Hopkins, Department of Psychological and Brain Sciences, USA

Fu Shih-Chien, National Taipei University of Education, Department of Counseling Psychology, Taipei, Taiwan

Du Li, Tongji University, Department of Research and Education, Shanghai Center for Women's and Children's Health, Shanghai, China

Zhu LiPing Tongji University, Department of Research and Education, Shanghai Center for Women's and Children's Health, Shanghai, China

Huang, Hsuan-Ying, The Chinese University of Hong Kong, Department of Anthropology, Hong Kong

Ewa Andersson, Karolinska Institutet, Department of Women's and Children's Health, Division of Reproductive Health, Stockholm, Sweden

Ganesh Acharya, Karolinska Institutet, Department of Clinical Science Intervention and Technology (CLINTEC), Division of Obstetrics and Gynecology, Stockholm, Sweden, Center for Fetal Medicine, Department of Obstetrics and Gynecology, Karolinska University Hospital, Stockholm, Sweden, Women´s Health and Perinatology Research Group, Department of Clinical Medicine, UiT-The Arctic University of Norway, Tromsø, Norway

*** Corresponding author: Schwank Simone; e-mail: simone.schwank@ki.se; phone: +46-707999574; Address: Karolinska Institutet, CLINTEC, University Hosptial, K56 Huddinge, 141 86 Stockholm, Sweden**

For peer review only - http://bmjopen.bmj.com/site/about/guidelines.xhtml

Abstract

Introduction

Mental health disorders are common during pregnancy and postnatal period, and can have serious adverse effects on women and their children. The consequences for global mental health due to COVID-19, are likely to be significant and may have a long-term impact on the global burden of disease. Besides physical vulnerability, pregnant women are at increased risk of mental health problems, such as anxiety, depression, and post-traumatic stress disorder (PTSD), due to the consequences of social distancing resulting in altered healthcare routines, less support from the family and friends, and in some cases, partners not being allowed to be present during prenatal visits, labor and delivery. Pregnant women may feel insecure and worried about the effects of COVID-19 on their unborn child if they get infected during pregnancy. Today, young urban women are used to utilizing internet services frequently and efficiently. Therefore, providing mental health support to pregnant women via internet may be effective in ameliorating their anxiety/depression, reducing the risk of serious mental health disorders, and lead to improved maternal and perinatal outcomes.

Overarching Aim

Our aim is to explore the effectiveness of a web-based psychosocial peer-to-peer support intervention in reducing the risk and severity of perinatal mental health disorders and preventing adverse pregnancy outcomes among pregnant women living in metropolitan urban settings.

Methods and Analysis

We plan to conduct a multi-center prospective randomized controlled trial, **M**ental health of **U**rban **M**others trial (MUM-Trial), in Hong Kong and Shanghai. Pregnant women will be recruited via internet-based application through nonprofit organisations. The women who consent will be randomized to receive a web-based peer-to-peer support intervention or usual care. Data will be analysed to identify the effect of intervention on EPDS and GAD7 scores and pregnancy outcomes. Impact of COVID-19 pandemic on maternal stress will be assesed using IES-R. Any differences in outcomes between cities will be addressed in subgroup analysis.

Ethics and dissemination

The study will be conducted according to the principles of Good Clinical Practice and will follow the ethical principles of the Declaration of Helsinki. The study protocol has been approved by the ethical review board of Chinese University Hong Kong IRB #2019-8170, and Shanghai Center for Women's and Children's Health with IRB #2020-F001-12. The results will be disseminated at national and international scientific conferences, published in peer-reviewed medical journals, and spread to the public through social media, news outlets and podcasts.

Key Words: Perinatal depression, Anxiety, PTSD, Pregnancy, Web-based intervention, Psychosocial Support, RCT, COVID-19

For peer review only

Strengths and limitations of this study

+ A evidence-based "*Thinking Healthy*" intervention will be applied using state-of-the-art eHealth peer-to-peer support to elucidate the role of internet-based virtual consultation and psycho-social support on maternal mental health.

+ Our multi-professional research team will ensure ethical standards, and the quality of intervention, data collection and follow-up.

+ An external data monitoring committee will monitor the trial and data quality.

- There is a risk of non-differential loss to follow up in both groups due to the long follow-up time.

- Due to the nature of intervention, investigators as well as the study participant will not be blinded to the intervention.

-Different cultures and healthcare systems in different study locations may have differential impacts on pregnancy outcomes.

For peer review only

Introduction

Pregnancy is a period of transition and great change, which may make some women more vulnerable to mental health problems. It is known that depressive symptoms during pregnancy may influence birth outcomes (1-3). The prevalence of postpartum depression (PPD) in Chinese women is similar to Western populations, with a prevalence of 10-20% (4-6). The prevalence of PPD in women of the one-child generation is reported to be higher compared to previous generations, particularly among women giving birth to a daughter (7). Prevalence rates are highest in the rural areas´ prefecture-level cities (25.4%), lower in the provincial capitals (19.5%), and lowest in the municipalities directly under central government (12.9%) (8, 9). There are substantial gaps in the accessibility of care (2). This rise in prevalence of PPD is expected to increase, as a result of the COVID-19 pandemic (10). Public health services should be aware of the higher risk of PPD among primipara (11, 12). Beyond effects on the mother alone, perinatal mental health issues can have long-term effects on child mental and physical health, behavior and cognition (13-17). Women often express feelings of inadequacy in the new mothering role, which can negatively impact their mental health and the relationship to their infant (18).

Effective treatments are needed to address high global rates of PPD with onset typically within 4 weeks after delivery (19) and maternal depression up to two years after delivery (20, 21). Web-based psychosocial support provided by trained public health nurses is an effective treatment method for PPD (22). Limited public health resources challenge the access to these services, which is why the current project uses web-based psychosocial support. With regards to risk perception, perceiving to be above the average total risk for woman herself or her infant increases the levels of uncertainty (23). Programs aimed at reducing PPD could achieve the most cost-efficient results by focusing the efforts in the critical time periods around childbirth (20).

For peer review only - http://bmjopen.bmj.com/site/about/guidelines.xhtml

During a crisis situation, such as the COVID-19 pandemic, pregnant women are even more vulnerable than the general population.(24) Pregnant women's distress may affect the fetus and is known to induce epigenetic changes in the placental genes (25). Increased risk of psychopathology is observed in children exposed to maternal prenatal distress, and elevated maternal cortisol and epigenetic regulation of placental glucocorticoid-pathway genes are potential mechanisms (26). Mothers who developed posttraumatic stress disorder (PTSD) in response to the 9/11 terrorist attacks had lower morning and evening salivary cortisol levels, compared to mothers who did not develop PTSD (27). Similar results were found among adult offspring of Holocaust survivors and those exposed to SARS and MERS outbreaks (28-30). Although the offspring of Holocaust survivors did not experience more traumatic events, they had a greater prevalence of current and lifetime PTSD and other psychiatric diagnoses than the demographically similar comparison subjects (31). These findings suggest that mothers with a greater susceptibility for developing PTSD may produce infants who are more easily distressed by novelty than infants of mothers who are less prone to develop PTSD (27, 32).

Previous Research

Screening programs appear to be effective in identifying pregnant women at risk of developing perinatal mental health disorders (33), but they are neither universally implemented in public healthcare nor necessarily lead to effective interventions. One reason for the limited effectiveness is the small number of multidisciplinary perinatal mental healthcare teams that provide for the assessment and treatment of perinatal mental health disorders (34). Pregnant women in Shanghai reported the lack of access to resources and a strong preference for web-based psychosocial support. Previous studies suggested that women who received the peer support intervention were at half the risk of developing postnatal depression at 12 weeks postpartum than those in the control group (35, 36).

There are challenges associated with supporting a family and raising children for young people living in modern metropolitan urban areas which may have a negative effect on maternal mental health. In Shanghai, China, a previous RCT at the Obstetrics and Gynecology Hospital of Fudan University concluded that prenatal evidence-based psychosocial intervention model for high-risk pregnant women holds potential as a preventive program that can address maternal health and improve birth outcomes (37). The mental health statuses of 6024 pregnant women were investigated when they registered for prenatal care from September 2015 to August 2016, where 23.89% and 9.44% had symptoms of depression and anxiety respectively (37, 38). There is a significant positive association between parity, depression, and older age and risk of developing PPD (11, 39). A cross-sectional study of 842 pregnant women with obstetric complication attending the same hospital, reported that the prevalence of major and minor depression in high-risk pregnant women during antenatal period was 8.3% and 28.9%, respectively (39).

Young couples in Hong Kong appear to have dificulties in balacing their traditional family roles and expectations with the hardships of livining in a modern society, as a recent study has found high levels of perinatal anxiety in families due to financial burden caused by having children and honoring the tradition. Having children is highly encouraged and a sign of prosperity in a traditional Chinese family, yet it is very expensive to raise even one child in Hong Kong (40). High level of work-family conflict is a strong predictor of mental health problems across pregnancy. Family-work balance was found to be notoriously poor in Hong Kong families, due to extremely high demands of working hours. Dual-earner families in Hong Kong Chinese parents indicated that work-family conflict was negatively related to job and life satisfaction.

For peer review only - http://bmjopen.bmj.com/site/about/guidelines.xhtml

The study showed that the coping behaviors of Hong Kong employed parents were largely ineffective to solve problems related to work-family conflict (41).

Objectives

Purpose and aims

Our aim is to explore the effectiveness of a web-based psychosocial peer-to-peer support intervention in reducing the risk and severity of perinatal mental health disorders and preventing adverse pregnancy outcomes among pregnant women residing in metropolitan urban settings.

Primary objective: To investigate the impact of a web-based psychosocial intervention on EPDS and GAD 7 scores in urban women living in Hong Kong and Shanghai.

Secondary objectives:

1. To explore the impact of web-based psychosocial intervention on mental health and pregnancy outcomes among women from different socio-economic and cultural environments.
2. To explore the relationship between mental health status of pregnant women and rates of elective cesarean section on maternal request.
3. To explore the impact COVID-19 pandemic and maternal stress levels based on IES-R.

Material and Methods

Trial design and setting

The MUM-Trial is a prospective randomized controlled trial that will be performed in order to assess the effect of psychosocial support during pregnancy provided by trained peers (intervention) vs. standard care (control group) focused on factors that can reduce mental health problems and adverse outcomes in pregnant and postpartum women. Participating women will be recruited from two large urban areas, i.e. Hong Kong (7.5 million inhabitants and among all maternity hospitals 11 027 annual deliveries (42), and Shanghai (25 million inhabitants and Shanghai Maternity and Child Health Care Hospital with more than117 700 deliveries per year). The intervention group will receive web-based psychosocial peer-to-peer support **2 times during the antenatal period** before 36 gestational weeks (at 18-22 weeks and 24-28 weeks) and the control group will receive usual standard antenatal care. Mental health status will be assessed using standard questionnaires, i.e. EPDS and GAD7 and IES-R. Information on the course and outcome of pregnancy will be obtained by sending a questionnaire at 4-6 weeks postnatally via a web-survey.

Hypothesis Feelings of acute stress, anxiety, depression, and more severe negative emotions such as PTSD that may lead to adverse pregnancy outcomes can be ameliorated by an appropriate internet-based psychosocial intervention started early in pregnancy, which in turn influences the women’s overall mental wellbeing and improves pregnancy outcomes.

Recruitment and blinding

For peer review only - http://bmjopen.bmj.com/site/about/guidelines.xhtml

Pregnant women in Hong Kong and Shanghai will be recruited via nonprofit organization websites in each country. Via web-link, the women will be guided to the research project website. The peer support website includes information about the project and informed consent. By completing the initial sociodemographic survey, the women can consent to participate in the research study. Then they will asked to use an app to follow the study and complete the survey questionnaires. The participating women will be automatically randomized to intervention (peer-to-peer web-based psychosocial support) or control group (standard care). Regardless of recruitment to intervention or control group, the women will continue their regular visits at the maternity clinics as per usual.
Both the women and the peer will be forbidden to inform anyone in the study about which study group they belonged to. However, due to the nature of intervention, investigators as well as the study participant will not be blinded to the intervention.

Inclusion criteria: Pregnant women between 12 and 18 weeks of gestation with viable intrauterine pregnancy
Exclusion criteria: History of severe psychiatric or substance abuse disorder requiring medical treatment, or presence of fetal chromosomal/structural abnormality.
Recruitment pathway: The participant recruitment pathway, the follow-up during pregnancy are described in Figure 1.

Intervention

Web-based psychosocial peer-to-peer support during pregnancy. Intervention will be administered using WhatsApp and WeChat APP to contact pregnant women and provide psychosocial support over chat, call, and video function.
The evidence-based eHealth peer-to-peer psychosocial intervention *"Thinking Healthy"*, will be tested in this RCT. In line with the World Health Organization's mhGAP Intervention Guide (mhGAP-IG), *"Thinking Healthy"* is used to identify and manage perinatal mental health conditions (distress, symptoms of depression, and anxiety) in non-specialized psychosocial support setting (43). *"Thinking Healthy"* includes guidance on evidence-based interventions to identify and manage a number of priority mental health conditions. One of the priority conditions is perinatal depression in the perinatal period. *"Thinking Healthy"* uses strategies (plans and activities) taken from internet-based cognitive behavioral therapy (ICBT) (44) to bring about a change in the mothers' symptoms and functioning. The training does not turn the non-specialized health workers or trained peers into CBT therapists – it only allows them to apply these strategies in their day-to-day work with women in the perinatal period. Non-specialized health workers or trained peers can practice *"Thinking Healthy"* (45). The web-based psychological support will be provided by trained peers using diverse web-based tools, incl. video calls, chat messaging, and voice calls for both women and their partners.

Intervention Group: Web-based psychosocial peer-to-peer support, using *"Thinking Healthy"* two times during pregnancy (see Figure 1).

Control Group: Standard perinatal care provided at maternity clinics.

Outcome measure

For peer review only - http://bmjopen.bmj.com/site/about/guidelines.xhtml

The primary outcome measure is the woman's EPDS and GAD7 scores assessed at 34-36 weeks of gestation and at 4-6 postnatally.

Secondary outcomes are: IES-R score, pregnancy complications including rate of preterm birth, occurrence of mental health disorders, rate of induction of labor, rate of vaginal birth, elective cesarean sections on maternal request, as well as other birth outcomes.

Psychological assessments

Edinburgh Postnatal Depression Scale (EPDS) (46, 47) is a widely validated screening instrument. A high EPDS score does not in itself confirm a diagnosis of depression. A score above a certain cut-off point may, however, indicate probable depressive disorder. In the present study, the EPDS will be administered in the third trimester of pregnancy (a 34-36 weeks) and at 4-6 weeks postpartum. An EPDS cut-off score of >10 has been found to be optimal for perinatal screening for depression (48).

The Generalized Anxiety Disorder (GAD7) questionnaire (49, 50) has been translated into Chinese and validated in general hospital outpatients, with a cut-off score of >10 (20) to indicate anxiety.

Impact Event Scale (IES-R) will be used to assess PTSD. It has been translated and validated Chinese (51-53) to assess symptoms of PTSD corresponding with DSM-IV. (51, 52) and a survey about COVID-19 will also be administered at the same time points (at 34-36 weeks of gestation and 4-6 weeks postpartum).

Sample size and power calculation

The sample size was calculated using an online sample size calculator (CinCalc.com) to detect a 20% difference in mean EPS scores between intervention vs. control groups with a 80% power and an alpha of 0,05 in each study location(54). EPDS scores show a normal distribution with a mean of 12.5 and standard deviation of 4.0 (55). Based on this, the calculated sample size is 40 participants in each arm (intervention/control) of the study in each study location (n=80 participants per city). Taking into account, possible dropouts, we estimated a required sample size of 100 participants for each city (study location), i.e. a total sample size of 200 participants.

Data management

Each study participant will receive a study number for identification. Data will be collected continuously and through the smartphone application entered into an electronic case report system. Data will be encrypted and safely stored on an electronic server at the Karolinska Institute in Stockholm, Sweden in compliance with European general data protection regulation (GDPR). Anonymized data will be extracted from the electronic case report system after data collection has ended and analyzed for the different endpoints.

Statistical Methods

Data will be analyzed per protocol using intention-to treat method. Average EPDS, GAD7 and IES-R scores will be compared between intervention and control groups using independent

For peer review only - http://bmjopen.bmj.com/site/about/guidelines.xhtml

sample t-test. Differences in proportions will be assessed using Chi-squared test. The non-normally distributed continuous variables, will be analyzed with the Mann-Whitney U test and the results of the intervention by Cohen's effect size. Multiple imputation will be used for missing data. The variables used in the imputation mode will be previous EPDS score, education level, and parity. All patients' demographic data, retrieved from web-survey, will be analyzed by descriptive (mean, median and range) statistics. For categorical variables, absolute frequency, percentages and/or proportions are calculated and Chi-squared or Fisher´s exact test will be used. A p-value of <0,05 will be considered significant. Subgroup analyses will be performed for several subgroups of EPDS scores. Spearman's correlation test will be used to test the correlation between maternal antenatal mental health at < 34-36 weeks EPDS, GAD7, IES-R, and at 6 weeks postpartum.

Analysis plan: The primary outcome (EPDS and GAD7 score) between intervention and control groups will be directly compared. We will also compare the proportions of women with EPDS score of >10 and >15 in two groups.

Confounders: Socio-economic status, educational level, parity, obstetric history including history of complications and trauma during previous pregnancies, number of surviving children, history of chronic diseases and use of medication, complications developing during current pregnancy (such as preeclampsia, gestational diabetes etc).

Patient and public involvement

No patients or public have been involved in the design, recruitment or conduction of the study.

Significance

Our study will generate evidence on whether web-based early intervention programs could be efficient in ameliorating the risk and severity of perinatal mental health disorders, and inform best clinical practice for women affected by the consequences of the COVID-19 pandemic.

The multi-center RCT to be conducted in Hong Kong and Shanghai will provide external validity and access to a larger sample size, as well as insight into socio-economic and cultural specifics of perinatal mental health disorders. Mental health disorders contribute up to 30% of the global burden of disease (56). Our RCT intends to provide insight into consequences of the COVID-19 pandemic on pregnant women's mental health status and pregnancy outcomes. It has already been reported that pregnant women experiencing PTSD are at great risk to pass on markers of PTSD to their unborn children through intergeneration transmission (57). Additionally, perinatal mental health disorders are associated with increased risk of psychological and developmental disturbances in children (58). Intergenerational transmission of maternal perinatal mental health disorders has shown to impact the child's physical and mental development (26, 58). These risks might be heightened in the time of the COVID-19 pandemic.

This interdisciplinary collaboration project may fill the research gap regarding the relationship between mental health and social environments. The study will raise awareness of mental health care and highlight the feasibility and value of web-based psychosocial support as a cost-effective way of preventing health perinatal mental health disorders in both mothers and their families during the COVID-19 pandemic.

For peer review only - http://bmjopen.bmj.com/site/about/guidelines.xhtml

Equipment and infrastructure Hong Kong's maternity hospitals and the Shanghai Maternity and Child Health Care Hospitals have about 11 027 and 117 700 respective deliveries annually. All the places have the necessary infrastructure to facilitate patient recruitment for research, conducting interviews, and distributing survey questions.

Ethics and dissemination

Adverse events

Peer to peer support are widely used for mental health among adolocent and women.

Research has demonstrated that peer supportive relationships help to contribute to positive adjustment and to buffer against stressors and adversities, including psychiatric problems (36).

No serious adverse effects are expected to occur as a result of our intervention, i.e. web-based psychological support. Any adverse event, defined as a serious undesirable experience/event or injury associated with the use of the study intervention will be reported to the health authorities of the respective study location and principal investigator. All participants will be covered by the national health insurance plans of the respective study sites.

Monitoring

The data monitoring committee for the RCT consists of Prof. Xu Biao, Karolinska Institute and Fudan University Dpt. Public Health, Prof. Mathias Allemand, University of Zurich, Dpt. Psychology, Prof. Sylvie Goldman, Columbia University, New York.

Dissemination plan

The study team plans to implement a knowledge translation using conventional methods for dissemination of scientific results but also modern communication strategies using social media. The information about the study will be spread through established collaborations with nonprofit organizations in in Hong Kong and Shanghai, via Chinese American Psychoanalytic Alliance (CAPA) and the Shanghai Center for Women's and Children's Health. We will use patient support groups, scientific and professional (midwifery, obstetric and psychological therapy) organizations to communicate about our study.

We will use established scientific channels to communicate with wider scientific community by publishing our protocol in an open access journal and findings by presenting at national and international scientific meetings and publishing in peer-reviewed international scientific journal We will also use more modern social media to disseminate our findings to the public.

Discussion

This RCT will provide high-quality data about the effect of web-based psychosocial peer-to-peer intervention during pregnancy on maternal mental health.

For peer review only - http://bmjopen.bmj.com/site/about/guidelines.xhtml

A recent review by Howard et al. (59) has shown that psychological and psychosocial interventions could be effective treatments for postnatal depression. Some studies from low-income and middle-income countries have shown that these can be provided effectively by trained peers or non-specialist workers (60). Evidence on the treatment of antenatal depression is limited to small trials (with 36–53 women) of interpersonal therapy, culturally relevant brief interpersonal psychotherapy, and CBT (61-63). Little research however exists about the effectiveness of such interventions (including web-based) for perinatal mental health problems (59).

The information generated by this project will also provide the fundament for public health policies to be put in place and resource allocation for supporting pregnant and postpartum women suffering from depression, anxiety, and PTSD, as a result of the COVID-19 pandemic. The implementation of a culturally sensitive maternal mental health education, addresses the need for more awareness of mental health issues in antenatal care. Our RCT can fill a knowledge gap and provide information on effectiveness of antenatal interventions provided by trained peers. Several studies have shown the effect of antenatal maternal mental health problems on child outcomes postpartum (64-66). Social and emotional support reduces associations between postnatal depression and early cognitive development in the child (67). The lack of recognition of perinatal mental health, distress, and disorders may have serious implications, and negative long-term effects on both mother and infant (66, 68). Therefore, it is important to recognize the transgenerational effect of perinatal mental health disorders and intervene early to avoid negative outcomes for the child.

The study's assessment of women's mental health status at the end of the pregnancy provides information on the expected benefits of psychosocial support during antenatal care, with emphasis on cost-effective web-based psychosocial peer-to-peer support, as an important alternative antenatal care model. Two recent studies have shown that as many as three out of four pregnant women use a pregnancy related application and find these applications beneficial and trustworthy (69, 70). This is likely to be even more relevant in urban dwellers.

The routines in perinatal mental health screening policies share similarities, yet they are still different in some aspects in Hong Kong and Shanghai. In Shanghai, first universal screening policies are put in place recently, i.e. at Shanghai Jiading District Maternity and Child Health Care Hospital and Obstetrics and Gynecology Hospital of Fudan University (71, 72). Currently, Shanghai provides limited universal screening for perinatal mental health problems, but lacks universal screening on provincial level, due to lack of resources. Adequate care for all the pregnant women with increased risk for developing perinatal mental health problems is currently not available Shanghai. In Hong Kong however, universal postpartum depression screening is one of the components of the Comprehensive Child Development Service (CCDS) in maternal and child health clinics, in collaboration with the Hospital Authority (HA) and Social Welfare Department (73). Further, Hong Kong women attending perinatal health care can get access to a webpage provided by the government, promoting public health information and raise awareness about perinatal depression and services provided for pregnant and postpartum women and their families (73). The resource allocation of universal screening and access to information is crucial, in order to reduce the burden of disease caused by perinatal mental health disorders on the family and the society.

For peer review only - http://bmjopen.bmj.com/site/about/guidelines.xhtml

As for most randomized trials, the external validity/ generalizability of our findings could be limited as we will target only urban dwelling population who are comfortable in using internet-based applications.

Declarations

Ethics approval and consent to participate

The study will be conducted according to the principles of Good Clinical Practice and has been approved by the ethical review boards the Chinese University Hong Kong (IRB #2019-8170), and Shanghai Center for Women's and Children's Health (IRB #2020-F001-12). Written consent is obtained from each participant before inclusion to the study. Participation in the study is voluntary and women are free to discontinue their participation at any time and for whatever reason without explanations.

All researchers directly involved in this RCT will have training and certification in Good Clinical Practice (GCP).

Availability of data and material

Anonymized data will be made available to researchers on reasonable request.

Authors' contributions

SES wrote the study protocol article with the support of GA and EA. SES and GA conceived the study and were responsible for formulating initial study design. All authors were involved in development of study design and take responsibility for the final study design. SES wrote the first draft of the study protocol. All authors have contributed to the protocol development and have read and approved the final protocol and this manuscript.

Funding

This project is funded by Swiss National Science Foundation SNSF (P2SKP3_187728). The funding agencies do not have any part in the design, data collection and analysis or reporting of this trial.

Competing interests

The authors declare that they have no competing interests.

Acknowledgements

For peer review only - http://bmjopen.bmj.com/site/about/guidelines.xhtml

The **Mental Health of Urban Mothers Trial (MUM-Trial)** in urban Hong Kong and Shanghai is supported by Karolinska Institute, Chinese University of Hong Kong, and Shanghai Government`s Center for Women's and Children's Health.
We would also like to acknowledge the nonprofit organizations for their support with patient recruitment.

Trial Registration and Status

Trial registration number NCT04363177

For peer review only

References

1. Dayan NJ, Creveuil NC, Marks NM, Conroy NS, Herlicoviez NM, Dreyfus NM, et al. Prenatal Depression, Prenatal Anxiety, and Spontaneous Preterm Birth: A Prospective Cohort Study Among Women With Early and Regular Care. Psychosomatic Medicine. 2006;68(6):938-46.
2. Siu Bonnie W, Leung Shirley S, Ip P, Hung S, O'Hara Michael W. Antenatal risk factors for postnatal depression: a prospective study of chinese women at maternal and child health centres. BMC Psychiatry. 2012;12(1):22.
3. Eberhard-Gran M. Depression in connection with parturition. Nedstemthet og depresjon i forbindelse med födsel. 2011;40(1).
4. Chan SWC, Levy V, Chung TKH, Lee D. A qualitative study of the experiences of a group of Hong Kong Chinese women diagnosed with postnatal depression. Journal of Advanced Nursing. 2002;39(6):571-9.
5. Chan SWC, Williamson V, McCutcheon H. A Comparative Study of the Experiences of a Group of Hong Kong Chinese and Australian Women Diagnosed With Postnatal Depression. Perspectives in Psychiatric Care. 2009;45(2):108-18.
6. Gao LL, Chan SWC, You L, Li X. Experiences of postpartum depression among first-time mothers in mainland China. Journal of Advanced Nursing. 2010;66(2):303-12.
7. Xiong R, Deng A, Wan B, Liu Y. Prevalence and factors associated with postpartum depression in women from single-child families. Int J Gynaecol Obstet. 2018;141(2):194-9.
8. Wong J, Fisher J. The role of traditional confinement practices in determining postpartum depression in women in Chinese cultures: A systematic review of the English language evidence. Journal of Affective Disorders. 2009;116(3):161-9.
9. Mu T-Y, Li Y-H, Pan H-F, Zhang L, Zha D-H, Zhang C-L, et al. Postpartum depressive mood (PDM) among Chinese women: a meta-analysis. Archives of Women's Mental Health. 2019;22(2):279-87.

For peer review only - http://bmjopen.bmj.com/site/about/guidelines.xhtml

10. Liu X, Kakade M, Fuller CJ, Fan B, Fang Y, Kong J, et al. Depression after exposure to stressful events: lessons learned from the severe acute respiratory syndrome epidemic. Comprehensive Psychiatry. 2012;53(1):15-23.
11. Glavin K, Smith L, Sørum R. Prevalence of postpartum depression in two municipalities in Norway. Scandinavian Journal of Caring Sciences. 2009;23(4):705-10.
12. Westgren M, Pettersson K, Hagberg H, Acharya G. Severe maternal morbidity and mortality associated with COVID-19: The risk should not be down-played. 2020.
13. Buss C, Davis EP, Hobel CJ, Sandman CA. Maternal pregnancy-specific anxiety is associated with child executive function at 6–9 years age. Stress. 2011;14(6):665-76.
14. Stein A, Netsi E, Lawrence PJ, Granger C, Kempton C, Craske MG, et al. Mitigating the effect of persistent postnatal depression on child outcomes through an intervention to treat depression and improve parenting: a randomised controlled trial. The Lancet Psychiatry. 2018;5(2):134-44.
15. Junge C, Garthus-Niegel S, Slinning K, Polte C, Simonsen TB, Eberhard-Gran M. The Impact of Perinatal Depression on Children's Social-Emotional Development: A Longitudinal Study.(Article). Maternal and Child Health Journal. 2017;21(3):607.
16. Loh CC, Vostanis P. Perceived mother-infant relationship difficulties in postnatal depression. Infant and Child Development. 2004;13(2):159-71.
17. Huang L, Acharya G. Novel corona virus disease (COVID-19) in pregnancy: What clinical recommendations to follow? Acta Obstetricia et Gynecologica Scandinavica. 2020:439-42.
18. Nelson AM. Transition to Motherhood. Oxford, UK2003. p. 465-77.
19. American Psychiatric A. Diagnostic and statistical manual of mental disorders : DSM-5. Fifth edition. ed: Arlington, VA : American Psychiatric Association; 2013.
20. R-H Xie G, He M, Walker SW, Wen SW. Postpartum Depression, Antenatal Social Support, And Postnatal Social Support. Annals of Epidemiology. 2008;18(9):736-.
21. Halbreich U, Karkun S. Cross-cultural and social diversity of prevalence of postpartum depression and depressive symptoms. Journal of Affective Disorders. 2006;91(2):97-111.
22. Glavin K, Smith L, Sørum R, Ellefsen B. Supportive counselling by public health nurses for women with postpartum depression. Journal of Advanced Nursing. 2010;66(6):1317-27.
23. Teitler JO, Plaza R, Hegyi T, Kruse L, Reichman NE. Elective Deliveries and Neonatal Outcomes in Full-Term Pregnancies. American journal of epidemiology. 2019;188(4):674.
24. WHO. Naming the coronavirus disease (COVID-19) and the virus that causes it. World Health Organization; 2020.
25. Monk C, Feng T, Lee S, Krupska I, Champagne FA, Tycko B. Distress During Pregnancy: Epigenetic Regulation of Placenta Glucocorticoid-Related Genes and Fetal Neurobehavior. American Journal of Psychiatry. 2016;173(7):705-13.
26. Doyle C, Werner E, Feng T, Lee S, Altemus M, Isler JR, et al. Pregnancy distress gets under fetal skin: Maternal ambulatory assessment & sex differences in prenatal development. Developmental Psychobiology. 2015;57(5):607-25.
27. Brand SR, Engel SM, Canfield RL, Yehuda R. The effect of maternal PTSD following in utero trauma exposure on behavior and temperament in the 9-month-old infant. Annals of the New York Academy of Sciences. 2006;1071:454.
28. Yoon M-K, Kim S-Y, Ko H-S, Lee M-S. System effectiveness of detection, brief intervention and refer to treatment for the people with post-traumatic emotional distress by MERS: a case report of community-based proactive intervention in South Korea.(middle

For peer review only - http://bmjopen.bmj.com/site/about/guidelines.xhtml

eastern respiratory syndrome)(Report). International Journal of Mental Health Systems. 2016;10(1).
29. Brooks KS, Dunn JR, Amlôt JR, Rubin JG, Greenberg JN. A Systematic, Thematic Review of Social and Occupational Factors Associated With Psychological Outcomes in Healthcare Employees During an Infectious Disease Outbreak. Journal of Occupational and Environmental Medicine. 2018;60(3):248-57.
30. Yehuda R, Engel SM, Brand SR, Seckl J, Marcus SM, Berkowitz GS. Transgenerational Effects of Posttraumatic Stress Disorder in Babies of Mothers Exposed to the World Trade Center Attacks during Pregnancy. The Journal of Clinical Endocrinology & Metabolism. 2005;90(7):4115-8.
31. Yehuda R, Schmeidler J, Wainberg M, Binder-Brynes K, Duvdevani T. Vulnerability to Posttraumatic Stress Disorder in Adult Offspring of Holocaust Survivors. American Journal of Psychiatry. 1998;155(9):1163-71.
32. Sprang G, Silman M. Posttraumatic stress disorder in parents and youth after health-related disasters. Disaster medicine and public health preparedness. 2013;7(1):105.
33. Wickberg B, Bendix M, Wetterholm MB, Skalkidou A. Perinatal mental health around the world: priorities for research and service development in Sweden. BJPsych International. 2019;17(1):1-3.
34. Schwank SE, Lindgren, H., Wickberg, B., Ding, Y., Andersson, E. Perinatal Mental Health in China: Views of key health system informants in Shanghai – A Qualitative Study. Public Health Journal. 2019;3:76-81.
35. Dennis CL, Hodnett E, Kenton L, Weston J, Zupancic J, Stewart DE, et al. Effect of peer support on prevention of postnatal depression among high risk women: multisite randomised controlled trial. BMJ. 2009;338(jan15 2).
36. Solomon P. Peer Support/Peer Provided Services Underlying Processes, Benefits, and Critical Ingredients. Psychiatric Rehabilitation Journal. 2004;27(4):392-401.
37. Zhao Y, Munro-Kramer ML, Shi S, Wang J, Luo J. A randomized controlled trial: effects of a prenatal depression intervention on perinatal outcomes among Chinese high-risk pregnant women with medically defined complications. Archives of women's mental health. 2017;20(2):333.
38. Qiao YX, Wang J, Li J, Ablat A. The prevalence and related risk factors of anxiety and depression symptoms among Chinese pregnant women in Shanghai. Australian and New Zealand Journal of Obstetrics and Gynaecology. 2009;49(2):185-90.
39. Zhao Y, Kane I, Mao L, Shi S, Wang J, Lin Q, et al. The Prevalence of Antenatal Depression and its Related Factors in Chinese Pregnant Women who Present with Obstetrical Complications. Archives of Psychiatric Nursing. 2016;30(3):316-21.
40. Koh YW, Lee AM, Chan CY, Fong DYT, Lee CP, Leung KY, et al. Survey on examining prevalence of paternal anxiety and its risk factors in perinatal period in Hong Kong: a longitudinal study. BMC public health. 2015;15(10732):1131.
41. Aryee S, Luk V, Leung A, Lo S. Role Stressors, Interrole Conflict, and Well-Being: The Moderating Influence of Spousal Support and Coping Behaviors among Employed Parents in Hong Kong. Journal of Vocational Behavior. 1999;54(2):259-78.
42. Kong GoH. Population. In: Department CaS, editor. Hong Kong2020.
43. Keynejad RC, Dua T, Barbui C, Thornicroft G. WHO Mental Health Gap Action Programme (mhGAP) Intervention Guide: a systematic review of evidence from low and middle-income countries. Evidence Based Mental Health. 2017;21(1).
44. Webb AC, Rosso MI, Rauch LS. Internet-Based Cognitive-Behavioral Therapy for Depression: Current Progress and Future Directions. Harvard Review of Psychiatry. 2017;25(3):114-22.

For peer review only - http://bmjopen.bmj.com/site/about/guidelines.xhtml

45. Organization WH. Thinking healthy: a manual for psychosocial management of perinatal depression. Geneva: WHO Press; 2015.
46. Cox JL, Holden JM, Sagovsky R. Detection of postnatal depression. Development of the 10- item Edinburgh Postnatal Depression Scale. The British journal of psychiatry : the journal of mental science. 1987;150:782.
47. Lau Y, Wang Y, Yin L, Chan KS, Guo X. Validation of the Mainland Chinese version of the Edinburgh Postnatal Depression Scale in Chengdu mothers. International Journal of Nursing Studies. 2010;47(9):1139-51.
48. Dugravier R, Tubach F, Saias T, Guedeney N, Pasquet B, Purper-Ouakil D, et al. Impact of a Manualized Multifocal Perinatal Home-Visiting Program Using Psychologists on Postnatal Depression: The CAPEDP Randomized Controlled Trial.(Research Article). PLoS ONE. 2013;8(8):e72216.
49. Spitzer RL, Kroenke K, Williams JBW, Löwe B. A Brief Measure for Assessing Generalized Anxiety Disorder: The GAD-7. Archives of Internal Medicine. 2006;166(10):1092-7.
50. Tong X, An D, McGonigal A, Park S-P, Zhou D. Validation of the Generalized Anxiety Disorder-7 (GAD-7) among Chinese people with epilepsy. Epilepsy Research. 2016;120:31-6.
51. Wu K, Chan S. Psychometric Properties of the Chinese Version of the Impact of Event Scale-Revised. Hong Kong J Psychiatry. 2004;14(4):2-8.
52. Creamer M, Bell R, Failla S. Psychometric properties of the Impact of Event Scale—Revised. Behaviour Research and Therapy. 2003;41(12):1489-96.
53. Sveen J, Low A, Dyster-Aas J, Ekselius L, Willebrand M, Gerdin B. Validation of a Swedish version of the Impact of Event Scale-Revised (IES-R) in patients with burns. Journal of Anxiety Disorders. 2010;24(6):618-22.
54. 2020 [cited 09.04.2020]. Available from: https://clincalc.com/stats/samplesize.aspx.
55. Smith-Nielsen J, Matthey S, Lange T, Vaever MS. Validation of the Edinburgh Postnatal Depression Scale against both DSM-5 and ICD-10 diagnostic criteria for depression.(Report). BMC Psychiatry. 2018;18(1).
56. Charlson FJ, Baxter AJ, Cheng HG, Shidhaye R, Whiteford HA. The burden of mental, neurological, and substance use disorders in China and India: a systematic analysis of community representative epidemiological studies. The Lancet. 2016;388(10042):376-89.
57. Bowers ME, Yehuda R. Intergenerational Transmission of Stress in Humans. Neuropsychopharmacology : official publication of the American College of Neuropsychopharmacology. 2016;41(1):232.
58. Stein A, Pearson RM, Goodman SH, Rapa E, Rahman A, McCallum M, et al. Effects of perinatal mental disorders on the fetus and child. The Lancet. 2014;384(9956):1800-19.
59. Howard LM, Piot P, Stein A. No health without perinatal mental health. The Lancet. 2014;384(9956):1723-4.
60. Patel V, Thornicroft G. Packages of Care for Mental, Neurological, and Substance Use Disorders in Low- and Middle-Income Countries: PLoS Medicine Series. PLoS Medicine. 2009;6(10):e1000160.
61. Dennis CL, Ross LE, Grigoriadis S. Psychosocial and psychological interventions for treating antenatal depression. The Cochrane database of systematic reviews. 2007(3):CD006309.
62. Grote N, Bledsoe S. Predicting Postpartum Depressive Symptoms in New Mothers: The Role of Optimism and Stress Frequency during Pregnancy. Health & Social Work. 2007;32(2):107-18.

For peer review only - http://bmjopen.bmj.com/site/about/guidelines.xhtml

63. Burns A, O'Mahen H, Baxter H, Bennert K, Wiles N, Ramchandani P, et al. A pilot randomised controlled trial of cognitive behavioural therapy for antenatal depression.(Clinical report). BMC Psychiatry. 2013;13(1).
64. Austin M-P. Marcé International Society position statement on psychosocial assessment and depression screening in perinatal women. Best Practice & Research Clinical Obstetrics & Gynaecology. 2014;28(1):179-87.
65. Milgrom J, Gemmill AW. Identifying Perinatal Depression and Anxiety : Evidence-Based Practice in Screening, Psychosocial Assessment and Management. Chicester: Chicester: John Wiley & Sons, Incorporated; 2015.
66. O'donnell KJ, Bugge Jensen A, Freeman L, Khalife N, O'connor TG, Glover V. Maternal prenatal anxiety and downregulation of placental 11β-HSD2. Psychoneuroendocrinology. 2012;37(6):818-26.
67. McManus B, Nugent J. A Neurobehavioral Intervention Incorporated into a State Early Intervention Program is Associated with Higher Perceived Quality of Care Among Parents of High-Risk Newborns. Official Publication of the National Council for Community Behavioral Healthcare. 2014;41(3):381-9.
68. Dunkel Schetter C, Tanner L. Anxiety, depression and stress in pregnancy: implications for mothers, children, research, and practice. Current Opinion in Psychiatry. 2012;25(2):141-8.
69. Osma J, Barrera AZ, Ramphos E. Are Pregnant and Postpartum Women Interested in Health-Related Apps? Implications for the Prevention of Perinatal Depression. Cyberpsychology, Behavior, and Social Networking. 2016;19(6):412-5.
70. Lupton D, Pedersen S. An Australian survey of women's use of pregnancy and parenting apps. Women and Birth. 2016;29(4):368-75.
71. Schwank S, Andersson E, Fu S-C, Wickberg B, Ding Y, Lindgren H. Care-seeking behavior and disclosure on self-reported mental health among young women in urban Shanghai, China. Health Psychology Open. 2019;Accepted.
72. Schwank S, Zhou Q, He Y, Acharya G. Perinatal mental health around the world: priorities for research and service development in China. British Journal of Psychiatry International. 2020;1(1):1-4.
73. Government HK. Non-Communicable Diseases Watch: Postpartum Depression: A Public Health Problem. In: Health P, editor. 2014.

For peer review only

Figure 1 – Overview of the study and time line

Year	*Jan-March*	*April-June*	*July-September*	*October-November*	*November-December*
2020	Protocol for survey study<br><br>IRB Hong Kong, Shanghai,<br><br>Set up Survey	**T1: First peer-to-peer intervention.** Participants recruitment. **(**200 participants for each city)<br>Randomization (antenatal < week 18).<br>Web-based Peer-to-peer support at 18-22 weeks	**T2: Second peer-to-peer intervention.** Web-based Peer-to-peer support at 24-28 weeks	**T3: post intervention** (34-36 weeks of gestation). Psychological tests: EPDS, GAD7, IES-R, COVID-19 19 survey	**T4: post intervention** (postpartum approx. 4-6 weeks after childbirth). Psychological tests: EPDS, GAD7, IES-R, COVID-19 survey
**2021**	Data collection completed	Analysis and interpretation of data	Report/manuscript writing	Reports/manuscript writing	Submission/Publication of results

For peer review only

BMJ Open

Mental Health of Urban Mothers (MUM) Study: A multi-center randomized controlled trial

Journal:	*BMJ Open*
Manuscript ID	bmjopen-2020-041133.R1
Article Type:	Protocol
Date Submitted by the Author:	01-Oct-2020
Complete List of Authors:	Schwank, Simone Eliane; Karolinska Institute, CLINTEC; UiT-The Arctic University of Norway, Tromsø, Norway, Women´s Health and Perinatology Research Group, Department of Clinical Medicine<br>Chung, Ho-Fung; The University of Hong Kong, Psychiatry<br>Hsu, Mandy; Johns Hopkins University, Psychological and Brain Sciences<br>Fu, Shih-Chien; National Taipei University of Education, Counseling Psychology<br>Du, Li; Tongji University, Department of Research and Education<br>Zhu, Liping; Tongji University, Department of Research and Education<br>Huang, Hsuan-Ying; The Chinese University Hong Kong, Anthropology<br>Andersson, Ewa; Karolinska Institutet, Department of Women´s and Children´s Health<br>Acharya, G; Karolinska Institutet; UiT-The Arctic University of Norway, Tromsø, Norway, Women´s Health and Perinatology Research Group, Department of Clinical Medicine,
**Primary Subject Heading**:	Global health
Secondary Subject Heading:	Mental health, Obstetrics and gynaecology, Public health, Health policy
Keywords:	Depression & mood disorders < PSYCHIATRY, Anxiety disorders < PSYCHIATRY, PERINATOLOGY, INFECTIOUS DISEASES, Reproductive medicine < GYNAECOLOGY

SCHOLARONE™
Manuscripts

I, the Submitting Author has the right to grant and does grant on behalf of all authors of the Work (as defined in the below author licence), an exclusive licence and/or a non-exclusive licence for contributions from authors who are: i) UK Crown employees; ii) where BMJ has agreed a CC-BY licence shall apply, and/or iii) in accordance with the terms applicable for US Federal Government officers or employees acting as part of their official duties; on a worldwide, perpetual, irrevocable, royalty-free basis to BMJ Publishing Group Ltd ("BMJ") its licensees and where the relevant Journal is co-owned by BMJ to the co-owners of the Journal, to publish the Work in this journal and any other BMJ products and to exploit all rights, as set out in our licence.

The Submitting Author accepts and understands that any supply made under these terms is made by BMJ to the Submitting Author unless you are acting as an employee on behalf of your employer or a postgraduate student of an affiliated institution which is paying any applicable article publishing charge ("APC") for Open Access articles. Where the Submitting Author wishes to make the Work available on an Open Access basis (and intends to pay the relevant APC), the terms of reuse of such Open Access shall be governed by a Creative Commons licence – details of these licences and which Creative Commons licence will apply to this Work are set out in our licence referred to above.

Other than as permitted in any relevant BMJ Author's Self Archiving Policies, I confirm this Work has not been accepted for publication elsewhere, is not being considered for publication elsewhere and does not duplicate material already published. I confirm all authors consent to publication of this Work and authorise the granting of this licence.

For peer review only

Mental Health of Urban Mothers (MUM) Study: A multi-center randomized controlled trial

Simone Schwank*, Division of Obstetrics and Gynecology, Department of Clinical Science Intervention and Technology (CLINTEC), Karolinska Institutet, Stockholm, Sweden.
Women´s Health and Perinatology Research Group, Department of Clinical Medicine, UiT-The Arctic University of Norway, Tromsø, Norway

Chung, Ho-Fung, Department of Psychiatry, The University of Hong Kong, Hong Kong, China

Mandy Hsu, Department of Psychological and Brain Sciences Johns Hopkins University, Baltimore, USA

Fu Shih-Chien, Department of Counseling Psychology, National Taipei University of Education,Taipei, Taiwan

Du Li, Department of Research and Education, Tongji University and Shanghai Center for Women's and Children's Health, Shanghai, China

Zhu LiPing Department of Research and Education, Tongji University and Shanghai Center for Women's and Children's Health, Shanghai, China

Huang, Hsuan-Ying, Department of Anthropology, The Chinese University of Hong Kong Hong Kong, China

Ewa Andersson, Department of Women's and Children's Health, Division of Reproductive Health, Karolinska Institutet, Stockholm, Sweden

Ganesh Acharya, Division of Obstetrics and Gynecology, Department of Clinical Science Intervention and Technology (CLINTEC), Karolinska Institutet and Center for Fetal Medicine, Department of Obstetrics and Gynecology, Karolinska University Hospital, Stockholm, Sweden
Women´s Health and Perinatology Research Group, Department of Clinical Medicine, UiT-The Arctic University of Norway, Tromsø, Norway
ORCID ID: https://orcid.org/0000-0002-1997-3107

*** Corresponding author: Schwank Simone; e-mail: simone.schwank@ki.se; phone: +46-707999574; Address: Karolinska Institutet, CLINTEC, University Hosptial, K56 Huddinge, 141 86 Stockholm, Sweden**

For peer review only - http://bmjopen.bmj.com/site/about/guidelines.xhtml

Abstract

Introduction

Mental health disorders are common during pregnancy and postnatal period, and can have serious adverse effects on women and their children. The consequences for global mental health due to COVID-19 are likely to be significant and may have a long-term impact on the global burden of disease. Besides physical vulnerability, pregnant women are at increased risk of mental health problems, such as anxiety, depression, and post-traumatic stress disorder due to the consequences of social distancing resulting in altered healthcare routines, less support from the family and friends, and in some cases, partners not being allowed to be present during prenatal visits, labor and delivery. Higher than expected rates of of perinatal anxiety and depression have been already reported during the pandemic. Pregnant women may feel insecure and worried about the effects of COVID-19 on their unborn child if they get infected during pregnancy. Today, young urban women are used to utilizing internet services frequently and efficiently. Therefore, providing mental health support to pregnant women via internet may be effective in ameliorating their anxiety/depression reducing the risk of serious mental health disorders, and lead to improved maternal and perinatal outcomes.

Overarching Aim

Our aim is to explore the effectiveness of a web-based psychosocial peer-to-peer support intervention in reducing the risk and severity of perinatal mental health disorders and preventing adverse pregnancy outcomes among pregnant women living in metropolitan urban settings.

Methods and Analysis

We plan to conduct a multi-center prospective randomized controlled trial, **M**ental health of **U**rban **M**others trial (MUM-Trial), in Hong Kong and Shanghai. Pregnant women will be recruited via internet-based application through nonprofit organisations. The women who consent will be randomized to receive a web-based peer-to-peer support intervention or usual care. Data will be analyzed to identify the effect of intervention on EPDS and GAD7 scores and pregnancy outcomes. Impact of COVID-19 pandemic on maternal stress will be assesed using IES-R. Any differences in outcomes between cities will be addressed in subgroup analysis.

Ethics and dissemination

The study will be conducted according to the principles of Good Clinical Practice and will follow the ethical principles of the Declaration of Helsinki. The study protocol has been approved by the ethical review board of Chinese University of Hong Kong (IRB #2019-8170) and Shanghai Center for Women's and Children's Health with (IRB #2020-F001-12). The results will be disseminated at national and international scientific conferences, published in peer-reviewed medical journals, and spread to the public through social media, news outlets and podcasts.

For peer review only - http://bmjopen.bmj.com/site/about/guidelines.xhtml

Key Words: Anxiety, COVID-19, Perinatal depression, Pregnancy, Psychosocial Support, RCT, Web-based intervention.

Strengths and limitations of this study

+ A evidence-based "*Thinking Healthy"* intervention will be applied using state-of-the-art eHealth peer-to-peer support to elucidate the role of internet-based virtual consultation and psycho-social support on maternal mental health.

+ Our multi-professional research team will ensure ethical standards, and the quality of intervention, data collection and follow-up.

+ An external data monitoring committee will monitor the trial and data quality.

- Due to the nature of intervention, investigators as well as the study participants will not be blinded to the intervention.

-Different cultures and healthcare systems in different study locations may have differential impacts on pregnancy outcomes.

For peer review only

Introduction

Pregnancy is a period of transition and great change, which may make some women more vulnerable to mental health problems. It is known that depressive symptoms during pregnancy may influence birth outcomes (1-3). The prevalence of postpartum depression (PPD) in Chinese women is similar to Western populations, with a prevalence of 10-20% (4-6). The prevalence of PPD in women of the one-child generation is reported to be higher compared to previous generations, particularly among women giving birth to a daughter (7). Prevalence rates are highest in the rural areas´ prefecture-level cities (25.4%), lower in the provincial capitals (19.5%), and lowest in the municipalities directly under central government (12.9%) (8, 9). Furthermore, there are substantial gaps in the accessibility of care (2). Perinatal mental health disorders may become more prevalent during a time of acute crisis, and prevalence of maternal anxiety, distress and PPD can be expected to increase in China and elsewhere as a result of the COVID-19 pandemic (10, 11). In this context, a recent study found that 35.4% of the participating women had an Edinburgh Postnatal Depression score (EPDS) >13 (12). However, there might be cross-national differences in risk factors and impact of pandemic on the prevalence of perinatal mental health disorders.

Some pregnant women might be predisposed to posttraumatic stress disorder (PTSD) during a crisis situation, such as the COVID-19 pandemic. Mothers who developed PTSD in response to the 9/11 terrorist attacks had lower morning and evening salivary cortisol levels, compared to mothers who did not develop PTSD (13). Beyond effects on the mother alone, perinatal mental health issues can have long-term effects on child´s mental and physical health, behavior and cognition (14-18). Pregnant women's distress may affect the fetus and is known to induce epigenetic changes in the placental genes (19). Increased risk of psychopathology is observed in children exposed to maternal prenatal distress, and elevated maternal cortisol and epigenetic regulation of placental glucocorticoid-pathway genes are potential mechanisms (20). Women

For peer review only - http://bmjopen.bmj.com/site/about/guidelines.xhtml

often express feelings of inadequacy in the new mothering role, which can negatively impact their mental health and the relationship to their infant (21).

Effective treatments are needed to address high global rates of PPD with onset typically within 4 weeks after delivery (22) and maternal depression up to two years after delivery (23, 24). Programs aimed at reducing PPD could achieve the most cost-efficient results by focusing the efforts in the critical time periods around childbirth (23).Web-based psychosocial support provided by trained public health nurses is an effective treatment method for PPD (25). Limited public health resources challenge the access to mental health services, that is why use of web-based psychosocial support could be an alternative. Women perceive the risk for themselves or their infants to be above average during global crises which increase the levels of uncertainty. However, face to face consultations during a pandemic are likely to increase the risk of disease transmission. Therefore, easily accessible eHealth support could provide fast and resource-effective care during the COVID-19 pandemic.

Previous Research

Screening programs appear to be effective in identifying pregnant women at risk of developing perinatal mental health disorders (26), but they are neither universally implemented in public healthcare nor necessarily lead to effective interventions. One reason for the limited effectiveness is the small number of multidisciplinary perinatal mental healthcare teams that provide for the assessment and treatment of perinatal mental health disorders (27). Pregnant women in Shanghai reported the lack of access to resources and a strong preference for web-based psychosocial support. Previous studies suggested that women who received the peer support intervention were at half the risk of developing postnatal depression at 12 weeks postpartum than those in the control group (28, 29). A recent systematic review has shown that therapist-supported internet-based cognitive behavaroral therapy (iCBT) significantly improves stress, anxiety, and depressive symptoms among postpartum although the magnitude of effect wa variable (30).

There are challenges associated with supporting a family and raising children for young people living in modern metropolitan urban areas which may have a negative effect on maternal mental health. In Shanghai, China, a previous RCT at the Obstetrics and Gynecology Hospital of Fudan University concluded that prenatal evidence-based psychosocial intervention model for high-risk pregnant women holds potential as a preventive program that can address maternal health and improve birth outcomes (31). The mental health statuses of 6024 pregnant women were investigated when they registered for prenatal care from September 2015 to August 2016, where 23.89% and 9.44% had symptoms of depression and anxiety respectively (31, 32). There is a significant positive association between parity, depression, and older age and risk of developing PPD (33, 34). A cross-sectional study of 842 pregnant women with obstetric complication attending the same hospital, reported that the prevalence of major and minor depression in high-risk pregnant women during antenatal period was 8.3% and 28.9%, respectively (33).

Young couples in Hong Kong appear to have dificulties in balacing their traditional family roles and expectations with the hardships of living in a modern society, as a recent study has found high levels of perinatal anxiety in families due to financial burden caused by having children

and honoring the tradition. Having children is highly encouraged and a sign of prosperity in a traditional Chinese family, yet it is very expensive to raise even one child in Hong Kong (35). High level of work-family conflict is a strong predictor of mental health problems across pregnancy. Family-work balance was found to be notoriously poor in Hong Kong families, due to extremely high demands of working hours. Dual-earner families in Hong Kong Chinese parents indicated that work-family conflict was negatively related to job and life satisfaction. The study showed that the coping behaviors of Hong Kong employed parents were largely ineffective to solve problems related to work-family conflict (36).

Objectives

Purpose and aims
Our aim is to explore the effectiveness of a web-based psychosocial peer-to-peer support intervention in reducing the risk and severity of perinatal mental health disorders and preventing adverse pregnancy outcomes among pregnant women residing in metropolitan urban settings.

Primary objective: To investigate the impact of a web-based psychosocial intervention on EPDS among urban pregnant women living in Hong Kong and Shanghai.

Secondary objectives:
1. To investigate the impact of a web-based psychosocial intervention on maternal anxiety levels assessed by GAD7 scores.
2. To explore the impact of web-based psychosocial intervention on mental health and pregnancy outcomes among women from different socio-economic and cultural environments.
3. To explore the relationship between mental health status of pregnant women and rates of elective cesarean section on maternal request.
4. To explore the impact COVID-19 pandemic on maternal stress levels assessed by IES-R.

Material and Methods

Trial design and setting

The MUM-Trial is a prospective randomized controlled trial that will be performed in order to assess the effect of psychosocial support during pregnancy provided by trained peers (intervention) vs. standard care (control group) focused on factors that can reduce mental health problems and adverse outcomes in pregnant and postpartum women. Participating women will be recruited from two large urban areas, i.e. Hong Kong (7.5 million inhabitants and among all maternity hospitals 11 027 annual deliveries (37), and Shanghai (25 million inhabitants and Shanghai Maternity and Child Health Care Hospital with more than117 700 deliveries per year). The intervention group will receive web-based psychosocial peer-to-peer support **2 times during the antenatal period** before 36 gestational weeks (at 18-22 weeks and 24-28 weeks) and the control group will receive usual standard antenatal care. Mental health status will be assessed using standard questionnaires, i.e. EPDS and GAD7 and IES-R. Information on the course and outcome of pregnancy will be obtained by sending a questionnaire at 4-6 weeks postnatally via a web-survey.

Hypothesis Feelings of acute stress, anxiety, depression, and more severe negative emotions such as PTSD that may lead to adverse pregnancy outcomes can be ameliorated by an

For peer review only - http://bmjopen.bmj.com/site/about/guidelines.xhtml

appropriate internet-based psychosocial intervention started early in pregnancy, which in turn influences the women's overall mental wellbeing and improves pregnancy outcomes.

Recruitment and blinding

Pregnant women in Hong Kong and Shanghai will be recruited via nonprofit organization websites in each country. Via web-link, the women will be guided to the research project website. The peer support website includes information about the project and informed consent. By completing the initial sociodemographic survey, the women can consent to participate in the research study. Then they will asked to use an app to follow the study and complete the survey questionnaires. The participating women will be automatically randomized to intervention (peer-to-peer web-based psychosocial support) or control group (standard care). Regardless of recruitment to intervention or control group, the women will continue their regular visits at the maternity clinics as per usual.

Both the women and the peer will be forbidden to inform anyone in the study about which study group they belonged to. However, due to the nature of intervention, investigators as well as the study participant will not be blinded to the intervention.

Inclusion criteria: Pregnant women between 12 and 18 weeks of gestation with viable intrauterine pregnancy

Exclusion criteria: History of severe psychiatric or substance abuse disorder requiring medical treatment, or presence of fetal chromosomal/structural abnormality.

Recruitment pathway: The participant recruitment pathway, the follow-up during pregnancy are described in Figure 1.

Intervention

Web-based psychosocial peer-to-peer support during pregnancy. Intervention will be administered using WhatsApp and WeChat APP to contact pregnant women and provide psychosocial support over chat, call, and video function.

The evidence-based eHealth peer-to-peer psychosocial intervention *"Thinking Healthy"*, will be tested in this RCT. In line with the World Health Organization's mhGAP Intervention Guide (mhGAP-IG), *"Thinking Healthy"* is used to identify and manage perinatal mental health conditions (distress, symptoms of depression, and anxiety) in non-specialized psychosocial support setting (38). *"Thinking Healthy"* includes guidance on evidence-based interventions to identify and manage a number of priority mental health conditions. *"Thinking Healthy"* has been widely used in low and low-middle income countries with limited mental health professionals (39). One of such priority conditions is perinatal depression. *"Thinking Healthy"* uses strategies (plans and activities) taken from iCBT(40) to bring about a change in the mothers' symptoms and functioning. The training does not turn the non-specialized health workers or trained peers into cognitive behavioral therapists – it only allows them to apply these strategies in their day-to-day work with women in the perinatal period. Non-specialized health workers or trained peers can practice *"Thinking Healthy"* (41). In our RCT, the web-based psychological support will be provided by trained peers using diverse web-based tools, including video calls, chat messaging, and voice calls for both women and their partners.

For peer review only - http://bmjopen.bmj.com/site/about/guidelines.xhtml

Intervention Group: Web-based psychosocial peer-to-peer support, using *"Thinking Healthy"* two times during pregnancy (see Figure 1).

Control Group: Standard perinatal care provided at maternity clinics.

Outcome measures

The primary outcome measure is the woman's EPDS assessed at 34-36 weeks of gestation and at 4-6 weeks postnatally.

Secondary outcomes are: GAD7 scores to assess maternal anxiety levels, IES-R score to assess PTSD, pregnancy complications including occurrence of mental health disorders, rate of preterm birth, rate of induction of labor, rate of vaginal birth, elective cesarean sections on maternal request, as well as other birth outcomes.

Psychological assessments
Edinburgh Postnatal Depression Scale (EPDS) (42, 43) is a widely validated screening instrument. A high EPDS score does not in itself confirm a diagnosis of depression. A score above a certain cut-off point may, however, indicate probable depressive disorder. In the present study, the EPDS will be administered in the third trimester of pregnancy (a 34-36 weeks) and at 4-6 weeks postpartum. An EPDS cut-off score of >10 has been found to be optimal for perinatal screening for depression (44).

The Generalized Anxiety Disorder (GAD7) questionnaire (45, 46) has been translated into Chinese and validated in general hospital outpatients, with a cut-off score of >10 (23) to indicate anxiety.

Impact Event Scale (IES-R) will be used to assess PTSD. It has been translated and validated Chinese (47-49) to assess symptoms of PTSD corresponding with DSM-IV. (47, 48) and a survey about COVID-19 will also be administered at the same time points (at 34-36 weeks of gestation and 4-6 weeks postpartum).

The EPDS, GAD7, and IES-R scales were chosen over psychodiagnositc interviews, as they show high reliability and validity and are more cost-effective and efficient comparing to psychodiagnostic interviews (50).

Sample size and power calculation

The sample size was calculated using an online sample size calculator (CinCalc.com) to detect a 20% difference in mean EPDS scores between intervention vs. control groups with a 80% power and an alpha of 0.05 in each study location (51). EPDS scores show a normal distribution with a mean of 12.5 and standard deviation of 4.0 (52). A difference of 20% in primary outcome is of clinical relevance with regards to the symptomatology of PPD (53). Based on this, the calculated sample size is 40 participants in each arm (intervention/control) of the study in each study location (n=80 participants per city). Taking into account, possible dropouts, we estimated a required sample size of 100 participants for each city (study location), i.e. a total sample size of 200 participants.

For peer review only - http://bmjopen.bmj.com/site/about/guidelines.xhtml

Figure 1 – Overview of the study and time line
About here

Data management

Each study participant will receive a study number for identification. Data will be collected continuously and through the smartphone application entered into an electronic case report system. Data will be encrypted and safely stored on an electronic server at the Karolinska Institute in Stockholm, Sweden in compliance with European general data protection regulation (GDPR). Anonymized data will be extracted from the electronic case report system after data collection has ended and analyzed for the different endpoints.

Statistical Methods

Data will be analyzed per protocol using intention-to treat method. Average EPDS, GAD7 and IES-R scores will be compared between intervention and control groups and across time points (prenatal and postnatal), using independent sample t-test. Differences in proportions will be assessed using Chi-squared test. The non-normally distributed continuous variables, will be analyzed with the Mann-Whitney U test and the results of the intervention by Cohen's effect size. Multiple imputation will be used for missing data. The variables used in the imputation mode will be previous EPDS score, education level, and parity. All patients' demographic data, retrieved from web-survey, will be analyzed by descriptive (mean, median and range) statistics. For categorical variables, absolute frequency, percentages and/or proportions will be calculated and Chi-squared or Fisher´s exact test will be used for compsrision between groups. For the primary outcome measure a two-sided p-value of <0.05 will be considered significant. Subgroup analyses will be performed for several subgroups of EPDS scores. Spearman's correlation test will be used to test the correlation between maternal antenatal mental health characteristics assessed by EPDS, GAD7 and IES-R at < 34-36 weeks and at 6 weeks postpartum.
Analysis plan: The EPDS , GAD7 score and IES-R between intervention and control groups will be directly compared. We will also compare the proportions of women with EPDS score of >10 and >15 in two groups.
Confounders: Socio-economic status, educational level, parity, obstetric history including history of complications and trauma during previous pregnancies, number of surviving children, history of chronic diseases and use of medication, complications developing during current pregnancy (such as preeclampsia, gestational diabetes etc).

Patient and public involvement
No patients or public have been involved in the design, recruitment or conduction of the study.

Significance

Our study will generate evidence on whether web-based early intervention programs could be efficient in ameliorating the risk and severity of perinatal mental health disorders, and inform best clinical practice for women affected by the consequences of the COVID-19 pandemic.

For peer review only - http://bmjopen.bmj.com/site/about/guidelines.xhtml

This multi-center RCT to be conducted in Hong Kong and Shanghai is expected provide access to an adequate sample size and external validity, as well as insight into socio-economic and cultural specifics of perinatal mental health disorders. Mental health disorders contribute up to 30% of the global burden of disease (54) and their prevalence is expected by rise during the COVID-19 pandemic. Intergenerational transmission of maternal perinatal mental health disorders has been shown to impact the child's physical and mental development (20, 55). These risks might be heightened in the time of the COVID-19 pandemic. Our RCT intends to provide some insight into consequences of the COVID-19 pandemic on pregnant women's mental health status and pregnancy outcomes.

This interdisciplinary collaboration project may fill the research gap regarding the relationship between mental health and social environments. The study will raise awareness of mental health care and highlight the feasibility and value of web-based psychosocial support as a cost-effective way of preventing/amiolderating mental health disorders in mothers and their families during the COVID-19 pandemic.

Equipment and infrastructure Hong Kong's maternity hospitals and the Shanghai Maternity and Child Health Care Hospitals have about 11 027 and 117 700 respective deliveries annually. All the places have the necessary infrastructure to facilitate patient recruitment for research, conducting interviews, and distributing survey questions.

Ethics and dissemination

Adverse events

Peer to peer support is widely used for mental health promotion among adolocent and women. Previous research has demonstrated that peer supportive relationships help to contribute to positive adjustment and to buffer against stressors and adversities, including psychiatric problems (29).

No serious adverse effects are expected to occur as a result of our intervention, i.e. web-based psychological support. Any adverse event, defined as a serious undesirable experience/event or injury associated with the use of the study intervention will be reported to the health authorities of the respective study location and principal investigator. All participants will be covered by the national health insurance plans of the respective study sites.

Monitoring

The data monitoring committee for the RCT consists of Prof. Xu Biao, Karolinska Institutet, Stockholm, Sweden and Department of Public Health, Fudan University, Shanghai, China, Prof. Mathias Allemand, Department of Psychology, University of Zurich, Switzerland, and Prof. Sylvie Goldman, Columbia University, New York, USA.

Dissemination plan

The study team plans to implement a knowledge translation using conventional methods for dissemination of scientific results but also modern communication strategies using social

For peer review only - http://bmjopen.bmj.com/site/about/guidelines.xhtml

media. The information about the study will be spread through established collaborations with nonprofit organizations in in Hong Kong and Shanghai, via Chinese American Psychoanalytic Alliance (CAPA) and the Shanghai Center for Women's and Children's Health. We will use patient support groups, scientific and professional (midwifery, obstetric and psychological therapy) organizations to communicate about our study.

We will use established scientific channels to communicate with wider scientific community by publishing our protocol in an open access journal and findings by presenting at national and international scientific meetings and publishing in peer-reviewed international scientific journal We will also use more modern social media to disseminate our findings to the public.

Discussion

This RCT will provide high-quality data about the effect of web-based psychosocial peer-to-peer intervention during pregnancy on maternal mental health. A recent review by Howard et al. (56) has shown that psychological and psychosocial interventions could be effective treatments for postnatal depression. Some studies from low- and middle-income countries have shown that these treatments can be provided effectively by trained peers or non-specialist workers (57). Evidence on the treatment of antenatal depression is limited to small trials (with 36–53 women) of interpersonal therapy, culturally relevant brief interpersonal psychotherapy, and CBT (58-60). Little research however exists about the effectiveness of such interventions (including web-based) for other perinatal mental health problems (56).

The information generated by this project will provide the fundament for public health policies to be put in place and resource allocation for supporting pregnant and postpartum women suffering from depression, anxiety, and PTSD, as a result of the COVID-19 pandemic.

The implementation of a culturally sensitive maternal mental health education, addresses the need for more awareness of mental health issues in antenatal care. Our RCT can fill a knowledge gap and provide information on effectiveness of antenatal interventions provided by trained peers. Several studies have shown the effect of antenatal maternal mental health problems on child outcomes postpartum (61-63). Social and emotional support reduces associations between postnatal depression and early cognitive development in the child (64). The lack of recognition of perinatal mental health, distress, and disorders may have serious implications, and negative long-term effects on both mother and infant (63, 65). Therefore, it is important to recognize the transgenerational effect of perinatal mental health disorders and intervene early to avoid negative outcomes for the child.

The study's assessment of women's mental health status at the end of the pregnancy provides information on the expected benefits of psychosocial support during antenatal care, with emphasis on cost-effective web-based psychosocial peer-to-peer support, as an important alternative care model. Two recent studies have shown that as many as three out of four

For peer review only

pregnant women use a pregnancy related internet application and find these applications beneficial and trustworthy (66, 67). This is likely to be even more relevant in urban dwellers.

The routines in perinatal mental health screening policies share similarities, yet they are still different in some aspects in Hong Kong and Shanghai. In Shanghai, first universal prenatal health screening policies are put in place recently, i.e. at Shanghai Jiading District Maternity and Child Health Care Hospital and Obstetrics and Gynecology Hospital of Fudan University (68, 69). Currently, Shanghai provides only limited universal screening for perinatal mental health problems, but lacks universal screening on provincial level. Adequate care for all the pregnant women with increased risk for developing perinatal mental health problems is currently not available in Shanghai. In Hong Kong, however, universal postpartum depression screening is one of the components of the Comprehensive Child Development Service (CCDS) in maternal and child health clinics, in collaboration with the Hospital Authority (HA) and Social Welfare Department (70). Further, Hong Kong women attending perinatal health care can get access to a webpage provided by the government, promoting public health information and raise awareness about perinatal depression and services provided for pregnant and postpartum women and their families (70). The resource allocation for universal screening and access to information might be crucial, in order to reduce the burden of disease caused by perinatal mental health disorders on the family and the society. Our study will allow to compare the impact of intervention in two different healthcare settings.

As for most randomized trials, the external validity/ generalizability of our findings could be limited as we will target only urban dwelling population who are comfortable in using internet-based applications.

Declarations

Ethics approval and consent to participate

The study will be conducted according to the principles of Good Clinical Practice and has been approved by the ethical review boards the Chinese University Hong Kong (IRB #2019-8170), and Shanghai Center for Women's and Children's Health (IRB #2020-F001-12). Written consent is obtained from each participant before inclusion to the study. Participation in the study is voluntary and women are free to discontinue their participation at any time and for whatever reason without explanations.

All researchers directly involved in this RCT will have training and certification in Good Clinical Practice (GCP).

Availability of data and material

Anonymized data will be made available to researchers on reasonable request.

Authors' contributions

For peer review only - http://bmjopen.bmj.com/site/about/guidelines.xhtml

SES wrote the study protocol article with the support of GA and EA. SES and GA conceived the study and were responsible for formulating initial study design together with HH local PI in Hong Kong responsible for conducting the study in Hong Kong, CHF is responsible for recruitment of participants and set up all epidemiological background to imitate the study in Hong Kong and Shanghai. ZLP is the local PI in Shanghai and together with DL responsible for running the study in Shanghai. MH was contributed to writing the protocol and conduct a literature search. FSC is responsible for recruitment of participants. All authors were involved in development of study design and take responsibility for the final study design. SES wrote the first draft of the study protocol. All authors have contributed to the protocol development and have read and approved the final protocol and this manuscript.

Funding

This project is funded by Swiss National Science Foundation SNSF (P2SKP3_187728). The funding agencies do not have any part in the design, data collection and analysis or reporting of this trial.

Competing interests

The authors declare that they have no competing interests.

Acknowledgements

The **Mental Health of Urban Mothers Trial (MUM-Trial)** in urban Hong Kong and Shanghai is supported by Karolinska Institutet, Chinese University of Hong Kong, and Shanghai Government`s Center for Women's and Children's Health.
We would also like to acknowledge the nonprofit organizations for their support with patient recruitment.

Trial Registration and Status

Trial registration number NCT04363177

Figure 1 – Overview of the study and time line

For peer review only

References

1. Dayan NJ, Creveuil NC, Marks NM, Conroy NS, Herlicoviez NM, Dreyfus NM, et al. Prenatal Depression, Prenatal Anxiety, and Spontaneous Preterm Birth: A Prospective Cohort Study Among Women With Early and Regular Care. Psychosomatic Medicine. 2006;68(6):938-46.
2. Siu Bonnie W, Leung Shirley S, Ip P, Hung S, O'Hara Michael W. Antenatal risk factors for postnatal depression: a prospective study of chinese women at maternal and child health centres. BMC Psychiatry. 2012;12(1):22.
3. Eberhard-Gran M. Depression in connection with parturition. Nedstemthet og depresjon i forbindelse med födsel. 2011;40(1).
4. Chan SWC, Levy V, Chung TKH, Lee D. A qualitative study of the experiences of a group of Hong Kong Chinese women diagnosed with postnatal depression. Journal of Advanced Nursing. 2002;39(6):571-9.
5. Chan SWC, Williamson V, McCutcheon H. A Comparative Study of the Experiences of a Group of Hong Kong Chinese and Australian Women Diagnosed With Postnatal Depression. Perspectives in Psychiatric Care. 2009;45(2):108-18.
6. Gao LL, Chan SWC, You L, Li X. Experiences of postpartum depression among first-time mothers in mainland China. Journal of Advanced Nursing. 2010;66(2):303-12.
7. Xiong R, Deng A, Wan B, Liu Y. Prevalence and factors associated with postpartum depression in women from single-child families. Int J Gynaecol Obstet. 2018;141(2):194-9.
8. Wong J, Fisher J. The role of traditional confinement practices in determining postpartum depression in women in Chinese cultures: A systematic review of the English language evidence. Journal of Affective Disorders. 2009;116(3):161-9.
9. Mu T-Y, Li Y-H, Pan H-F, Zhang L, Zha D-H, Zhang C-L, et al. Postpartum depressive mood (PDM) among Chinese women: a meta-analysis. Archives of Women's Mental Health. 2019;22(2):279-87.
10. Cameron EE, Joyce KM, Delaquis CP, Reynolds K, Protudjer JLP, Roos LE. Maternal psychological distress & mental health service use during the COVID-19 pandemic. Journal of affective disorders. 2020;276:765-74.
11. Wu Y, Zhang C, Liu H, Duan C, Li C, Fan J, et al. Perinatal depressive and anxiety symptoms of pregnant women during the coronavirus disease 2019 outbreak in China. American journal of obstetrics and gynecology. 2020;223(2):240.e1-.e9.
12. Durankuş F, Aksu E. Effects of the COVID-19 pandemic on anxiety and depressive symptoms in pregnant women: a preliminary study. The journal of maternal-fetal & neonatal medicine. 2020:1-7.
13. Brand SR, Engel SM, Canfield RL, Yehuda R. The effect of maternal PTSD following in utero trauma exposure on behavior and temperament in the 9-month-old infant. Annals of the New York Academy of Sciences. 2006;1071:454.
14. Buss C, Davis EP, Hobel CJ, Sandman CA. Maternal pregnancy-specific anxiety is associated with child executive function at 6–9 years age. Stress. 2011;14(6):665-76.
15. Stein A, Netsi E, Lawrence PJ, Granger C, Kempton C, Craske MG, et al. Mitigating the effect of persistent postnatal depression on child outcomes through an intervention to treat depression and improve parenting: a randomised controlled trial. The Lancet Psychiatry. 2018;5(2):134-44.
16. Junge C, Garthus-Niegel S, Slinning K, Polte C, Simonsen TB, Eberhard-Gran M. The Impact of Perinatal Depression on Children's Social-Emotional Development: A Longitudinal Study.(Article). Maternal and Child Health Journal. 2017;21(3):607.
17. Loh CC, Vostanis P. Perceived mother-infant relationship difficulties in postnatal depression. Infant and Child Development. 2004;13(2):159-71.

For peer review only - http://bmjopen.bmj.com/site/about/guidelines.xhtml

18. Huang L, Acharya G. Novel corona virus disease (COVID-19) in pregnancy: What clinical recommendations to follow? Acta Obstetricia et Gynecologica Scandinavica. 2020:439-42.
19. Monk C, Feng T, Lee S, Krupska I, Champagne FA, Tycko B. Distress During Pregnancy: Epigenetic Regulation of Placenta Glucocorticoid-Related Genes and Fetal Neurobehavior. American Journal of Psychiatry. 2016;173(7):705-13.
20. Doyle C, Werner E, Feng T, Lee S, Altemus M, Isler JR, et al. Pregnancy distress gets under fetal skin: Maternal ambulatory assessment & sex differences in prenatal development. Developmental Psychobiology. 2015;57(5):607-25.
21. Nelson AM. Transition to Motherhood. Oxford, UK2003. p. 465-77.
22. American Psychiatric A. Diagnostic and statistical manual of mental disorders : DSM-5. Fifth edition. ed: Arlington, VA : American Psychiatric Association; 2013.
23. R-H Xie G, He M, Walker SW, Wen SW. Postpartum Depression, Antenatal Social Support, And Postnatal Social Support. Annals of Epidemiology. 2008;18(9):736-.
24. Halbreich U, Karkun S. Cross-cultural and social diversity of prevalence of postpartum depression and depressive symptoms. Journal of Affective Disorders. 2006;91(2):97-111.
25. Glavin K, Smith L, Sørum R, Ellefsen B. Supportive counselling by public health nurses for women with postpartum depression. Journal of Advanced Nursing. 2010;66(6):1317-27.
26. Wickberg B, Bendix M, Wetterholm MB, Skalkidou A. Perinatal mental health around the world: priorities for research and service development in Sweden. BJPsych International. 2019;17(1):1-3.
27. Schwank SE, Lindgren, H., Wickberg, B., Ding, Y., Andersson, E. Perinatal Mental Health in China: Views of key health system informants in Shanghai – A Qualitative Study. Public Health Journal. 2019;3:76-81.
28. Dennis CL, Hodnett E, Kenton L, Weston J, Zupancic J, Stewart DE, et al. Effect of peer support on prevention of postnatal depression among high risk women: multisite randomised controlled trial. BMJ. 2009;338(jan15 2).
29. Solomon P. Peer Support/Peer Provided Services Underlying Processes, Benefits, and Critical Ingredients. Psychiatric Rehabilitation Journal. 2004;27(4):392-401.
30. Lau Y, Htun TP, Wong SN, Tam WSW, Klainin-Yobas P. Therapist-Supported Internet-Based Cognitive Behavior Therapy for Stress, Anxiety, and Depressive Symptoms Among Postpartum Women: A Systematic Review and Meta-Analysis. Journal of medical Internet research. 2017;19(4):e138.
31. Zhao Y, Munro-Kramer ML, Shi S, Wang J, Luo J. A randomized controlled trial: effects of a prenatal depression intervention on perinatal outcomes among Chinese high-risk pregnant women with medically defined complications. Archives of women's mental health. 2017;20(2):333.
32. Qiao YX, Wang J, Li J, Ablat A. The prevalence and related risk factors of anxiety and depression symptoms among Chinese pregnant women in Shanghai. Australian and New Zealand Journal of Obstetrics and Gynaecology. 2009;49(2):185-90.
33. Zhao Y, Kane I, Mao L, Shi S, Wang J, Lin Q, et al. The Prevalence of Antenatal Depression and its Related Factors in Chinese Pregnant Women who Present with Obstetrical Complications. Archives of Psychiatric Nursing. 2016;30(3):316-21.
34. Glavin K, Smith L, Sørum R. Prevalence of postpartum depression in two municipalities in Norway. Scandinavian Journal of Caring Sciences. 2009;23(4):705-10.
35. Koh YW, Lee AM, Chan CY, Fong DYT, Lee CP, Leung KY, et al. Survey on examining prevalence of paternal anxiety and its risk factors in perinatal period in Hong Kong: a longitudinal study. BMC public health. 2015;15(10732):1131.

For peer review only - http://bmjopen.bmj.com/site/about/guidelines.xhtml

36. Aryee S, Luk V, Leung A, Lo S. Role Stressors, Interrole Conflict, and Well-Being: The Moderating Influence of Spousal Support and Coping Behaviors among Employed Parents in Hong Kong. Journal of Vocational Behavior. 1999;54(2):259-78.
37. Kong GoH. Population. In: Department CaS, editor. Hong Kong2020.
38. Keynejad RC, Dua T, Barbui C, Thornicroft G. WHO Mental Health Gap Action Programme (mhGAP) Intervention Guide: a systematic review of evidence from low and middle-income countries. Evidence Based Mental Health. 2017;21(1).
39. Patel V. The future of psychiatry in low- and middle-income countries. Psychol Med. 2009;39(11):1759-62.
40. Webb AC, Rosso MI, Rauch LS. Internet-Based Cognitive-Behavioral Therapy for Depression: Current Progress and Future Directions. Harvard Review of Psychiatry. 2017;25(3):114-22.
41. Organization WH. Thinking healthy: a manual for psychosocial management of perinatal depression. Geneva: WHO Press; 2015.
42. Cox JL, Holden JM, Sagovsky R. Detection of postnatal depression. Development of the 10- item Edinburgh Postnatal Depression Scale. The British journal of psychiatry : the journal of mental science. 1987;150:782.
43. Lau Y, Wang Y, Yin L, Chan KS, Guo X. Validation of the Mainland Chinese version of the Edinburgh Postnatal Depression Scale in Chengdu mothers. International Journal of Nursing Studies. 2010;47(9):1139-51.
44. Dugravier R, Tubach F, Saias T, Guedeney N, Pasquet B, Purper-Ouakil D, et al. Impact of a Manualized Multifocal Perinatal Home-Visiting Program Using Psychologists on Postnatal Depression: The CAPEDP Randomized Controlled Trial.(Research Article). PLoS ONE. 2013;8(8):e72216.
45. Spitzer RL, Kroenke K, Williams JBW, Löwe B. A Brief Measure for Assessing Generalized Anxiety Disorder: The GAD-7. Archives of Internal Medicine. 2006;166(10):1092-7.
46. Tong X, An D, McGonigal A, Park S-P, Zhou D. Validation of the Generalized Anxiety Disorder-7 (GAD-7) among Chinese people with epilepsy. Epilepsy Research. 2016;120:31-6.
47. Wu K, Chan S. Psychometric Properties of the Chinese Version of the Impact of Event Scale-Revised. Hong Kong J Psychiatry. 2004;14(4):2-8.
48. Creamer M, Bell R, Failla S. Psychometric properties of the Impact of Event Scale—Revised. Behaviour Research and Therapy. 2003;41(12):1489-96.
49. Sveen J, Low A, Dyster-Aas J, Ekselius L, Willebrand M, Gerdin B. Validation of a Swedish version of the Impact of Event Scale-Revised (IES-R) in patients with burns. Journal of Anxiety Disorders. 2010;24(6):618-22.
50. Chorpita BF, Nakamura BJ. Dynamic Structure in Diagnostic Structured Interviewing: A Comparative Test of Accuracy and Efficiency. Journal of Psychopathology and Behavioral Assessment. 2008;30(1):52-60.
51. 2020 [cited 09.04.2020]. Available from: https://clincalc.com/stats/samplesize.aspx.
52. Smith-Nielsen J, Matthey S, Lange T, Vaever MS. Validation of the Edinburgh Postnatal Depression Scale against both DSM-5 and ICD-10 diagnostic criteria for depression.(Report). BMC Psychiatry. 2018;18(1).
53. Dennis C-L. Psychosocial and psychological interventions for prevention of postnatal depression: systematic review. BMJ. 2005;331(7507):15.
54. Charlson FJ, Baxter AJ, Cheng HG, Shidhaye R, Whiteford HA. The burden of mental, neurological, and substance use disorders in China and India: a systematic analysis of community representative epidemiological studies. The Lancet. 2016;388(10042):376-89.

For peer review only - http://bmjopen.bmj.com/site/about/guidelines.xhtml

55. Stein A, Pearson RM, Goodman SH, Rapa E, Rahman A, McCallum M, et al. Effects of perinatal mental disorders on the fetus and child. The Lancet. 2014;384(9956):1800-19.
56. Howard LM, Piot P, Stein A. No health without perinatal mental health. The Lancet. 2014;384(9956):1723-4.
57. Patel V, Thornicroft G. Packages of Care for Mental, Neurological, and Substance Use Disorders in Low- and Middle-Income Countries: PLoS Medicine Series. PLoS Medicine. 2009;6(10):e1000160.
58. Dennis CL, Ross LE, Grigoriadis S. Psychosocial and psychological interventions for treating antenatal depression. The Cochrane database of systematic reviews. 2007(3):CD006309.
59. Grote N, Bledsoe S. Predicting Postpartum Depressive Symptoms in New Mothers: The Role of Optimism and Stress Frequency during Pregnancy. Health & Social Work. 2007;32(2):107-18.
60. Burns A, O'Mahen H, Baxter H, Bennert K, Wiles N, Ramchandani P, et al. A pilot randomised controlled trial of cognitive behavioural therapy for antenatal depression.(Clinical report). BMC Psychiatry. 2013;13(1).
61. Austin M-P. Marcé International Society position statement on psychosocial assessment and depression screening in perinatal women. Best Practice & Research Clinical Obstetrics & Gynaecology. 2014;28(1):179-87.
62. Milgrom J, Gemmill AW. Identifying Perinatal Depression and Anxiety : Evidence-Based Practice in Screening, Psychosocial Assessment and Management. Chicester: Chicester: John Wiley & Sons, Incorporated; 2015.
63. O'donnell KJ, Bugge Jensen A, Freeman L, Khalife N, O'connor TG, Glover V. Maternal prenatal anxiety and downregulation of placental 11β-HSD2. Psychoneuroendocrinology. 2012;37(6):818-26.
64. McManus B, Nugent J. A Neurobehavioral Intervention Incorporated into a State Early Intervention Program is Associated with Higher Perceived Quality of Care Among Parents of High-Risk Newborns. Official Publication of the National Council for Community Behavioral Healthcare. 2014;41(3):381-9.
65. Dunkel Schetter C, Tanner L. Anxiety, depression and stress in pregnancy: implications for mothers, children, research, and practice. Current Opinion in Psychiatry. 2012;25(2):141-8.
66. Osma J, Barrera AZ, Ramphos E. Are Pregnant and Postpartum Women Interested in Health-Related Apps? Implications for the Prevention of Perinatal Depression. Cyberpsychology, Behavior, and Social Networking. 2016;19(6):412-5.
67. Lupton D, Pedersen S. An Australian survey of women's use of pregnancy and parenting apps. Women and Birth. 2016;29(4):368-75.
68. Schwank S, Andersson E, Fu S-C, Wickberg B, Ding Y, Lindgren H. Care-seeking behavior and disclosure on self-reported mental health among young women in urban Shanghai, China. Health Psychology Open. 2019;Accepted.
69. Schwank S, Zhou Q, He Y, Acharya G. Perinatal mental health around the world: priorities for research and service development in China. British Journal of Psychiatry International. 2020;1(1):1-4.
70. Government HK. Non-Communicable Diseases Watch: Postpartum Depression: A Public Health Problem. In: Health P, editor. 2014.

For peer review only - http://bmjopen.bmj.com/site/about/guidelines.xhtml

Figure 1 – Overview of the study and time line

Year	*Jan-March*	*April-June*	*July-September*	*October-November*	*November-December*
2020	Protocol for survey study<br><br>IRB Hong Kong, Shanghai,<br><br>Set up Survey	**T1: First peer-to-peer intervention.** Participants recruitment. **(**200 participants for each city)<br>Randomization (antenatal < week 18).<br>Web-based Peer-to-peer support at 18-22 weeks	**T2: Second peer-to-peer intervention.** Web-based Peer-to-peer support at 24-28 weeks	**T3: post intervention** (34-36 weeks of gestation). Psychological tests: EPDS, GAD7, IES-R, COVID-19 19 survey	**T4: post intervention** (postpartum approx. 4-6 weeks after childbirth). Psychological tests: EPDS, GAD7, IES-R, COVID-19 survey
**2021**	Data collection completed	Analysis and interpretation of data	Report/manuscript writing	Reports/manuscript writing	Submission/Publication of results

For peer review only

BMJ Open

Mental Health of Urban Mothers (MUM) Study: A multi-center randomized controlled trial, study protocol

Journal:	*BMJ Open*
Manuscript ID	bmjopen-2020-041133.R2
Article Type:	Protocol
Date Submitted by the Author:	27-Oct-2020
Complete List of Authors:	Schwank, Simone Eliane; Karolinska Institute, CLINTEC; UiT-The Arctic University of Norway, Tromsø, Norway, Women´s Health and Perinatology Research Group, Department of Clinical Medicine<br>Chung, Ho-Fung; The University of Hong Kong, Psychiatry<br>Hsu, Mandy; Johns Hopkins University, Psychological and Brain Sciences<br>Fu, Shih-Chien; National Taipei University of Education, Counseling Psychology<br>Du, Li; Tongji University, Department of Research and Education<br>Zhu, Liping; Tongji University, Department of Research and Education<br>Huang, Hsuan-Ying; The Chinese University Hong Kong, Anthropology<br>Andersson, Ewa; Karolinska Institutet, Department of Women´s and Children´s Health<br>Acharya, G; Karolinska Institutet; UiT-The Arctic University of Norway, Tromsø, Norway, Women´s Health and Perinatology Research Group, Department of Clinical Medicine,
**Primary Subject Heading**:	Global health
Secondary Subject Heading:	Mental health, Obstetrics and gynaecology, Public health, Health policy
Keywords:	Depression & mood disorders < PSYCHIATRY, Anxiety disorders < PSYCHIATRY, PERINATOLOGY, INFECTIOUS DISEASES, Reproductive medicine < GYNAECOLOGY

SCHOLARONE™
Manuscripts

I, the Submitting Author has the right to grant and does grant on behalf of all authors of the Work (as defined in the below author licence), an exclusive licence and/or a non-exclusive licence for contributions from authors who are: i) UK Crown employees; ii) where BMJ has agreed a CC-BY licence shall apply, and/or iii) in accordance with the terms applicable for US Federal Government officers or employees acting as part of their official duties; on a worldwide, perpetual, irrevocable, royalty-free basis to BMJ Publishing Group Ltd ("BMJ") its licensees and where the relevant Journal is co-owned by BMJ to the co-owners of the Journal, to publish the Work in this journal and any other BMJ products and to exploit all rights, as set out in our licence.

The Submitting Author accepts and understands that any supply made under these terms is made by BMJ to the Submitting Author unless you are acting as an employee on behalf of your employer or a postgraduate student of an affiliated institution which is paying any applicable article publishing charge ("APC") for Open Access articles. Where the Submitting Author wishes to make the Work available on an Open Access basis (and intends to pay the relevant APC), the terms of reuse of such Open Access shall be governed by a Creative Commons licence – details of these licences and which Creative Commons licence will apply to this Work are set out in our licence referred to above.

Other than as permitted in any relevant BMJ Author's Self Archiving Policies, I confirm this Work has not been accepted for publication elsewhere, is not being considered for publication elsewhere and does not duplicate material already published. I confirm all authors consent to publication of this Work and authorise the granting of this licence.

For peer review only

Mental Health of Urban Mothers (MUM) Study: A multi-center randomized controlled trial, study protocol

Simone Schwank*, Division of Obstetrics and Gynecology, Department of Clinical Science Intervention and Technology (CLINTEC), Karolinska Institutet, Stockholm, Sweden.
Women´s Health and Perinatology Research Group, Department of Clinical Medicine, UiT-The Arctic University of Norway, Tromsø, Norway

Chung, Ho-Fung, Department of Psychiatry, The University of Hong Kong, Hong Kong, China

Mandy Hsu, Department of Psychological & Brain Sciences, Johns Hopkins University, Baltimore, USA

Fu Shih-Chien, Department of Counseling Psychology, National Taipei University of Education,Taipei, Taiwan

Du Li, Department of Research and Education, Tongji University and Shanghai Center for Women's and Children's Health, Shanghai, China

Zhu LiPing, Department of Research and Education, Tongji University and Shanghai Center for Women's and Children's Health, Shanghai, China

Huang, Hsuan-Ying, Department of Anthropology, The Chinese University of Hong Kong Hong Kong, China

Ewa Andersson, Department of Women's and Children's Health, Division of Reproductive Health, Karolinska Institutet, Stockholm, Sweden

Ganesh Acharya, Division of Obstetrics and Gynecology, Department of Clinical Science Intervention and Technology (CLINTEC), Karolinska Institutet and Center for Fetal Medicine, Department of Obstetrics and Gynecology, Karolinska University Hospital, Stockholm, Sweden
Women´s Health and Perinatology Research Group, Department of Clinical Medicine, UiT-The Arctic University of Norway, Tromsø, Norway
ORCID ID: https://orcid.org/0000-0002-1997-3107

*** Corresponding author: Schwank Simone; e-mail: simone.schwank@ki.se; phone: +46-707999574; Address: Karolinska Institutet, CLINTEC, University Hosptial, K56 Huddinge, 141 86 Stockholm, Sweden**

For peer review only - http://bmjopen.bmj.com/site/about/guidelines.xhtml

Abstract

Introduction

Mental health disorders are common during pregnancy and the postnatal period, and can have serious adverse effects on women and their children. The consequences for global mental health due to COVID-19 are likely to be significant and may have a long-term impact on the global burden of disease. Besides physical vulnerability, pregnant women are at increased risk of mental health problems, such as anxiety, depression, and post-traumatic stress disorder due to the consequences of social distancing. It can result in altered healthcare routines, less support from the family and friends, and in some cases, partners not being allowed to be present during prenatal visits, labor, and delivery. Higher than expected rates of perinatal anxiety and depression have been already reported during the pandemic. Pregnant women may also feel insecure and worried about the effects of COVID-19 on their unborn child if they get infected during pregnancy. Today, young urban women are used to utilizing internet services frequently and efficiently. Therefore, providing mental health support to pregnant women via internet may be effective in ameliorating their anxiety/depression, reducing the risk of serious mental health disorders, and lead to improved maternal and perinatal outcomes.

Overarching Aim

Our aim is to explore the effectiveness of a web-based psychosocial peer-to-peer support intervention in reducing the risk and severity of perinatal mental health disorders and preventing adverse pregnancy outcomes among pregnant women living in metropolitan urban settings.

Methods and Analysis

We plan to conduct a multi-center prospective randomized controlled trial, **M**ental health of **U**rban **M**others trial (MUM-Trial). Pregnant women living in large metropolitan cities will be recruited using internet-based application through nonprofit organizations´websites. The women who consent will be randomized to receive a web-based peer-to-peer support intervention or usual care. Data will be analyzed to identify the effects of intervention on EPDS and GAD7 scores, as well as pregnancy outcomes. The impact of the COVID-19 pandemic on maternal stress will be assesed using IES-R. Any differences in outcomes between cities will be addressed in subgroup analyses.

Ethics and dissemination

The study will be conducted according to the principles of Good Clinical Practice and will follow the ethical principles of the Declaration of Helsinki. The study protocol has been approved by the ethical review board of Chinese University of Hong Kong (IRB #2019-8170) and Shanghai Center for Women's and Children's Health (IRB #2020-F001-12). The results will be disseminated at national and international scientific conferences, published in peer-reviewed medical journals, and spread to the public through social media, news outlets, and podcasts.

For peer review only - http://bmjopen.bmj.com/site/about/guidelines.xhtml

Key Words: Anxiety, COVID-19, Perinatal depression, Pregnancy, Psychosocial Support, RCT, Web-based intervention.

Strengths and limitations of this study

+ A evidence-based "*Thinking Healthy"* intervention will be applied using state-of-the-art eHealth peer-to-peer support to elucidate the role of internet-based virtual consultation and psycho-social support on maternal mental health.

+ Our multi-professional research team will ensure ethical standards along with the quality of intervention, data collection, and follow-up.

+ An external data monitoring committee will monitor the trial and data quality.

- Due to the nature of intervention, investigators, as well as the study participants, will not be blinded to the intervention.

-Different cultures and healthcare systems in different study locations may have different impacts on pregnancy outcomes.

Introduction

Pregnancy is a period of transition and great change, which may make some women more vulnerable to mental health problems. It is known that depressive symptoms during pregnancy may influence birth outcomes (1-3). The prevalence of postpartum depression (PPD) is increasing globally but varies considerably between different countries and cultures (4, 5). In China, the prevalence of PPD in women of the one-child generation is reported to be higher compared to previous generations, particularly among women giving birth to a daughter (6). Prevalence rates are highest in the rural areas´ prefecture-level cities (25.4%), lower in the provincial capitals (19.5%), and lowest in the municipalities directly under central government (12.9%) (7, 8). Furthermore, there are substantial gaps in the accessibility of care (2). Perinatal mental health disorders may become more prevalent during a time of acute crisis, and the prevalence of maternal anxiety, distress, and PPD can be expected to increase in China and elsewhere as a result of the COVID-19 pandemic (9, 10). In this context, a recent study found that 35.4% of the participating women had an Edinburgh Postnatal Depression score (EPDS) >13 (11). However, there might be cross-national differences in the risk factors and impact of pandemic on the prevalence of perinatal mental health disorders.

Some pregnant women might be predisposed to posttraumatic stress disorder (PTSD) during a crisis situation, such as the COVID-19 pandemic. Mothers who developed PTSD in response to the 9/11 terrorist attacks had lower morning and evening salivary cortisol levels, compared to mothers who did not develop PTSD (12). Beyond effects on the mother alone, perinatal mental health issues can have long-term effects on child´s mental and physical health, as well as their behavior and cognition (13-17). Distress in pregnant women may affect the fetus and is known to induce epigenetic changes in the placental genes (18). Increased risk of psychopathology is observed in children exposed to maternal prenatal distress. Elevated maternal cortisol and epigenetic regulation of placental glucocorticoid-pathway genes are

For peer review only - http://bmjopen.bmj.com/site/about/guidelines.xhtml

potential mechanisms for these observations (19). Women often express feelings of inadequacy in the new mothering role, which can furthermore negatively impact their mental health and the relationship to their infant (20).

Effective treatments are needed to address high global rates of PPD with onset typically within 4 weeks after delivery (21) and maternal depression up to two years after delivery (22, 23). Programs aimed at reducing PPD could achieve the most cost-efficient results by focusing efforts in the critical time periods around childbirth (22).Web-based psychosocial support provided by trained public health nurses is an effective treatment method for PPD (24). Limited public health resources are challenges to the accessibility of mental health services, which is why the use of web-based psychosocial support could be an good alternative. Women perceive the risk for themselves or their infants to be above average during global crises, which increases the levels of uncertainty. However, face-to-face consultations during a pandemic are likely to increase the risk of disease transmission. Therefore, easily accessible eHealth support could provide fast and resource-effective care during the COVID-19 pandemic.

Previous Research

Screening programs appear to be effective in identifying pregnant women at risk of developing perinatal mental health disorders (25), but they are neither universally implemented in public healthcare nor necessarily lead to effective interventions. One reason for the limited effectiveness is the small number of multidisciplinary perinatal mental healthcare teams that provide for the assessment and treatment of perinatal mental health disorders (26). Pregnant women in Shanghai reported a lack of access to resources and a strong preference for web-based psychosocial support. Previous studies suggested that women who received a peer support intervention were at half the risk of developing postnatal depression at 12 weeks postpartum than those in a control group (27, 28). A recent systematic review has shown that therapist-supported internet-based cognitive behavaroral therapy (iCBT) significantly improves stress, anxiety, and depressive symptoms among postpartum women, although the magnitude of effect was variable (29).

There are challenges associated with supporting a family and raising children for young people living in modern metropolitan urban areas. These challenges may have a negative effect on maternal mental health. In Shanghai, China, a previous RCT at the Obstetrics and Gynecology Hospital of Fudan University concluded that a prenatal evidence-based psychosocial intervention model for high-risk pregnant women holds potential as a preventive program that can address maternal health and improve birth outcomes (30). The mental health statuses of 6024 pregnant women were investigated when they registered for prenatal care from September 2015 to August 2016, where 23.89% and 9.44% had symptoms of depression and anxiety respectively (30, 31). There is a significant positive association between parity, depression, older age, and risk of developing PPD (32, 33). A cross-sectional study of 842 pregnant women with obstetric complications attending the same hospital reported that the prevalence of major and minor depression in high-risk pregnant women during antenatal period was 8.3% and 28.9%, respectively (32).

Young couples in Hong Kong appear to have dificulties in balacing their traditional family roles and expectations with the hardships of living in a modern society. A recent study has found high levels of perinatal anxiety in families due to the financial burden caused by having children to honoring traditional family roles. Having children is highly encouraged and a sign of

For peer review only - http://bmjopen.bmj.com/site/about/guidelines.xhtml

prosperity in a traditional Chinese family, yet, it is very expensive to raise even one child in Hong Kong (34). Having a high level of work-family conflict is a strong predictor of mental health problems across pregnancy. Family-work balance was also found to be notoriously poor in Hong Kong families, due to the extremely high demands of working hours. Dual-earner families in Hong Kong indicated that the work-family conflict was negatively related to job and life satisfaction. The study showed that the coping behaviors of Hong Kong employed parents were largely ineffective in solving problems related to work-family conflicts (35).

Objectives

Purpose and aims

Our aim is to explore the effectiveness of a web-based psychosocial peer-to-peer support intervention in reducing the risk and severity of perinatal mental health disorders and preventing adverse pregnancy outcomes among pregnant women residing in metropolitan urban settings.

Primary objective: To investigate the impact of a web-based psychosocial intervention on EPDS among urban pregnant women living in Hong Kong and Shanghai

Secondary Objectives

1. To investigate the impact of a web-based psychosocial intervention on maternal anxiety levels assessed by GAD7 scores.
2. To explore the impact COVID-19 pandemic on maternal stress levels assessed by IES-R.
3. To explore the impact of web-based psychosocial intervention on mental health and pregnancy outcomes among women from different socio-economic and cultural environments.
4. To explore the relationship between mental health status of pregnant women and rates of elective cesarean section on maternal request.

Material and Methods

Trial design and setting

The MUM-Trial is a prospective randomized controlled trial that will be performed in order to assess the effects of psychosocial support during pregnancy that focuses on factors that can reduce mental health problems and adverse outcomes in pregnant and postpartum women (intervention), vs. standard care (control group).The intervention will be delivered by trained peers. Participating women will be recruited from two large urban areas, i.e. Hong Kong (7.5 million inhabitants and among all maternity hospitals 11 027 annual deliveries (36), and Shanghai (25 million inhabitants and Shanghai Maternity and Child Health Care Hospital with more than117 700 deliveries per year). The intervention group will receive web-based psychosocial peer-to-peer support **2 times during the antenatal period** before 36 gestational weeks (at 18-22 weeks and 24-28 weeks) and the control group will receive the usual standard

For peer review only - http://bmjopen.bmj.com/site/about/guidelines.xhtml

antenatal care. Mental health status will be assessed using standard questionnaires, i.e. EPDS and GAD7 and IES-R. Information on the course and outcome of pregnancy will be obtained by sending a questionnaire at 4-6 weeks postnatally via a web-survey.

Hypothesis Feelings of acute stress, anxiety, depression, and more severe negative emotions such as PTSD that may lead to adverse pregnancy outcomes can be ameliorated by an appropriate internet-based psychosocial intervention started early in pregnancy, which will in turn influence the women's overall mental wellbeing and improve pregnancy outcomes.

Recruitment and blinding

Pregnant women will be recruited via nonprofit organization websites in each country. In addition to Hong Kong and Shanghai, we aim to include additional urban locations, including Stockholm, Sweden in this multicenter study. Via web-link, the women will be guided to the research project website. The peer support website includes information about the project and informed consent. By completing the initial sociodemographic survey, the women can consent to participate in the research study. Then, they will asked to use an app to follow the study and complete the survey questionnaires. The participating women will be automatically randomized to intervention (peer-to-peer web-based psychosocial support) or control group (standard care) by the computer-generated random sequence. Regardless of recruitment to the intervention or control group, the women will continue their regular visits at the maternity clinics as usual.
Both the women and their peers will be forbidden to inform anyone in the study about which study group they belonged to. However, due to the nature of intervention, investigators, as well as the study participants, will not be blinded to the intervention.

Inclusion criteria: Pregnant women between 12 and 18 weeks of gestation with viable intrauterine pregnancy
Exclusion criteria: History of severe psychiatric or substance abuse disorder requiring medical treatment, or presence of fetal chromosomal/structural abnormality.
Recruitment pathway: The participant recruitment pathway and the follow-up during pregnancy are described in Figure 1.

Intervention

The intervention is a web-based psychosocial peer-to-peer support during pregnancy. It will be administered using WhatsApp and WeChat APP to contact pregnant women and provide psychosocial support over chat, call, and video function.
The evidence-based eHealth peer-to-peer psychosocial intervention *"Thinking Healthy"*, will be tested in this RCT. In line with the World Health Organization's mhGAP Intervention Guide (mhGAP-IG), *"Thinking Healthy"* is used to identify and manage perinatal mental health conditions (distress, symptoms of depression, and anxiety) in non-specialized psychosocial support setting (37). *"Thinking Healthy"* includes guidance on evidence-based interventions to identify and manage a number of priority mental health conditions. *"Thinking Healthy"* has been widely used in low and low-middle income countries with limited mental health professionals (38). One of such priority conditions is perinatal depression. *"Thinking Healthy"* also uses strategies (plans and activities) taken from iCBT(39) to bring about a change in the mothers' symptoms and functioning. The training does not turn the non-specialized health

For peer review only - http://bmjopen.bmj.com/site/about/guidelines.xhtml

workers or trained peers into cognitive behavioral therapists – it only allows them to apply these strategies in their day-to-day work with women in the perinatal period. Non-specialized health workers or trained peers can practice *"Thinking Healthy"* (40). The research team provides support for the non-specialized health care workers and peers. In our RCT, the web-based psychological support will be provided by trained peers using diverse web-based tools, including video calls, chat messaging, and voice calls for both women and their partners.

Intervention Group: They will receive a web-based psychosocial peer-to-peer support, using *"Thinking Healthy"* two times during pregnancy (see Figure 1).

Control Group: They will receive standard perinatal care provided at maternity clinics.

Outcome measures

The primary outcome measure is the woman's EPDS assessed at 34-36 weeks of gestation and at 4-6 weeks postnatally.

Secondary outcomes are: GAD7 scores to assess maternal anxiety levels, IES-R score to assess PTSD, and pregnancy complications, including occurrence of mental health disorders, rate of preterm birth, rate of induction of labor, rate of vaginal birth, elective cesarean sections on maternal request, as well as other birth outcomes.

Psychological assessments

Edinburgh Postnatal Depression Scale (EPDS) (41, 42) is a widely validated screening instrument. A high EPDS score does not in itself confirm a diagnosis of depression. A score above a certain cut-off point may, however, indicate probable depressive disorder. In the present study, the EPDS will be administered in the third trimester of pregnancy (a 34-36 weeks) and at 4-6 weeks postpartum. An EPDS cut-off score of >10 has been found to be optimal for perinatal screening for depression (43).

The Generalized Anxiety Disorder (GAD7) questionnaire (44, 45) has been translated into Chinese and validated in general hospital outpatients, with a cut-off score of >10 (22) to indicate anxiety.

Impact Event Scale (IES-R) will be used to assess PTSD. It has been translated and validated in Chinese (46-48) to assess symptoms of PTSD corresponding with DSM-IV. (46, 47). A survey about COVID-19 will also be administered at the same time points (at 34-36 weeks of gestation and 4-6 weeks postpartum).

The EPDS, GAD7, and IES-R scales were chosen over psychodiagnositc interviews because they show high reliability and validity, and are more cost-effective and efficient compared to psychodiagnostic interviews (49).

Sample size and power calculation

The sample size was calculated using an online sample size calculator (CinCalc.com) to detect a 20% difference in mean EPDS scores between ithe ntervention vs. control groups with a 80% power and an alpha of 0.05 in each study location (50). EPDS scores show a normal distribution

For peer review only

with a mean of 12.5 and standard deviation of 4.0 (51). A difference of 20% in the primary outcome is of clinical relevance with regards to the symptomatology of PPD (52). Based on this, the calculated sample size is 40 participants in each arm (intervention/control) of the study in each study location (n=80 participants per city). Taking into account and possible dropouts, we estimated a required sample size of 100 participants for each city (study location). The same principle will be applied if any additional study locations are added to this multicentre RCT.

Figure 1 – Overview of the study and time line

About here

Data management

Each study participant will receive a study number for identification. Data will be collected continuously and through the smartphone application entered into an electronic case report system. Data will be encrypted and safely stored on an electronic server at the Karolinska Institute in Stockholm, Sweden in compliance with European general data protection regulation (GDPR). Anonymized data will be extracted from the electronic case report system after data collection has ended and analyzed for the different endpoints.

Statistical Methods

Data will be analyzed per protocol using an intention-to treat method. Average EPDS, GAD7 and IES-R scores will be compared between the intervention and control groups and across time points (prenatal and postnatal), using independent sample t-tests. Differences in proportions will be assessed using Chi-squared test. The non-normally distributed continuous variables will be analyzed with the Mann-Whitney U test, and the results of the intervention will be analyzed by Cohen's effect size. Multiple imputations will be used for missing data. The variables used in the imputation mode will be: previous EPDS score, education level, and parity. All patients' demographic data, retrieved from web-survey, will be analyzed by descriptive (mean, median and range) statistics. For categorical variables, absolute frequency, percentages, and/or proportions will be calculated. Chi-squared or Fisher´s exact test will be used for compsrison between groups. For the primary outcome measure, a two-sided p-value of <0.05 will be considered significant. Subgroup analyses will be performed for several subgroups of EPDS scores. Spearman's correlation test will be used to test the correlation between maternal antenatal mental health characteristics assessed by EPDS, GAD7 and IES-R at < 34-36 weeks and at 6 weeks postpartum.

Analysis plan: The EPDS, GAD7, and IES-R scores between intervention and control groups will be directly compared. We will also compare the proportions of women with EPDS score of >10 and >15 in the two groups.

Confounders: Socio-economic status, educational level, parity, obstetric history including history of complications and trauma during previous pregnancies, number of surviving children, history of chronic diseases and use of medication, complications developing during current pregnancy (such as preeclampsia, gestational diabetes etc) may all be confounders.

Patient and public involvement

No patients or public have been involved in the design, recruitment or conduction of the study.

For peer review only - http://bmjopen.bmj.com/site/about/guidelines.xhtml

Significance

Our study will generate evidence on whether web-based early intervention programs could be efficient in ameliorating the risk and severity of perinatal mental health disorders, and inform best clinical practice for women affected by the consequences of the COVID-19 pandemic.

This multi-center RCT to be conducted in Hong Kong and Shanghai is expected provide access to an adequate sample size and external validity, as well as insight into socio-economic and cultural specifics of perinatal mental health disorders. Mental health disorders contribute up to 30% of the global burden of disease (53) and their prevalence is expected by rise during the COVID-19 pandemic. Intergenerational transmission of maternal perinatal mental health disorders has been shown to impact the child's physical and mental development (19, 54). These risks might be heightened in the time of the COVID-19 pandemic. Our RCT intends to provide some insight into consequences of the COVID-19 pandemic on pregnant women's mental health status and pregnancy outcomes.

This interdisciplinary collaboration project may fill the research gap regarding the relationship between mental health and social environments. The study will raise awareness of mental health care and highlight the feasibility and value of web-based psychosocial support as a cost-effective way of preventing/amiolderating mental health disorders in mothers and their families during the COVID-19 pandemic.

Equipment and infrastructure Hong Kong's maternity hospitals and the Shanghai Maternity and Child Health Care Hospitals have about 11 027 and 117 700 respective deliveries annually. All the places have the necessary infrastructure to facilitate patient recruitment for research, conducting interviews, and distributing survey questions.

Ethics and dissemination

Adverse events

Peer to peer support is widely used for mental health promotion among adolocent and women. Previous research has demonstrated that peer supportive relationships help to contribute to positive adjustment and to buffer against stressors and adversities, including psychiatric problems (28).

No serious adverse effects are expected to occur as a result of our intervention, i.e. web-based psychological support. Any adverse event, defined as a serious undesirable experience/event or injury associated with the use of the study intervention, will be reported to the health authorities of the respective study location and principal investigator. All participants will be covered by the national health insurance plans of the respective study sites.

Monitoring

For peer review only - http://bmjopen.bmj.com/site/about/guidelines.xhtml

The data monitoring committee for the RCT consists of Prof. Xu Biao, Karolinska Institutet, Stockholm, Sweden and Department of Public Health, Fudan University, Shanghai, China, Prof. Mathias Allemand, Department of Psychology, University of Zurich, Switzerland, and Prof. Sylvie Goldman, Columbia University, New York, USA.

Dissemination plan

The study team plans to implement a knowledge translation using conventional methods for dissemination of scientific results, as well as modern communication strategies such as social media. The information about the study will be spread through established collaborations with nonprofit organizations in in Hong Kong and Shanghai, via Chinese American Psychoanalytic Alliance (CAPA) and the Shanghai Center for Women's and Children's Health. We will use patient support groups, scientific and professional (midwifery, obstetric and psychological therapy), to communicate about our study.

We will use established scientific channels to communicate with the wider scientific community by publishing our protocol in an open access journal. We will also communicate our findings by presenting at national and international scientific meetings, along with publishing in a peer-reviewed international scientific journal.
We will also use more modern techniques, such as social media, to disseminate our findings to the public.

Discussion

This RCT will provide high-quality data about the effect of web-based psychosocial peer-to-peer intervention during pregnancy on maternal mental health. A recent review by Howard et al. (55) has shown that psychological and psychosocial interventions could be effective treatments for postnatal depression. Some studies from low- and middle-income countries have shown that these treatments can be provided effectively by trained peers or non-specialist workers (56). Evidence on the treatment of antenatal depression is limited to small trials (with 36–53 women) of interpersonal therapy, culturally relevant brief interpersonal psychotherapy, and CBT (57-59). Little research exists about the effectiveness of such interventions (including web-based) for other perinatal mental health problems (55).
The information generated by this project could provide the basis for public health policies to be put in place and resource allocation for supporting pregnant and postpartum women suffering from depression, anxiety, and PTSD, as a result of the COVID-19 pandemic.

The implementation of a culturally sensitive maternal mental health education addresses the need for more awareness of mental health issues in antenatal care. Our RCT can fill a knowledge gap and provide information on effectiveness of antenatal interventions provided by trained peers. Several studies have shown the effect of antenatal maternal mental health problems on child outcomes postpartum (60-62). Social and emotional support reduces associations between postnatal

For peer review only - http://bmjopen.bmj.com/site/about/guidelines.xhtml

depression and early cognitive development in the child (63). The lack of recognition of perinatal mental health, distress, and disorders may have serious implications for negative long-term effects on both mother and infant (62, 64). Therefore, it is important to recognize the transgenerational effect of perinatal mental health disorders and to intervene early to avoid negative outcomes for the child.

The study's assessment of women's mental health status at the end of the pregnancy provides information on the expected benefits of psychosocial support during antenatal care, with emphasis on cost-effective web-based psychosocial peer-to-peer support as an important alternative care model. Two recent studies have shown that as many as three out of four pregnant women use a pregnancy related internet application and find these applications beneficial and trustworthy (65, 66). This is likely to be even more relevant in urban dwellers.

The routines in perinatal mental health screening policies share similarities, yet they are still different in some aspects in Hong Kong and Shanghai. In Shanghai, first universal prenatal health screening policies have been put in place recently, i.e. at Shanghai Jiading District Maternity and Child Health Care Hospital and Obstetrics and Gynecology Hospital of Fudan University (67, 68). Currently, Shanghai provides only limited universal screening for perinatal mental health problems, and lacks universal screening on provincial level. Adequate care for all pregnant women with increased risk for developing perinatal mental health problems is currently not available in Shanghai. In Hong Kong, however, universal postpartum depression screening is one of the components of the Comprehensive Child Development Service (CCDS) in maternal and child health clinics, in collaboration with the Hospital Authority (HA) and Social Welfare Department (69). Further, Hong Kong women attending perinatal health care can get access to a webpage provided by the government, promoting the spread of public health information, as well as raised awarness about perinatal depression and services provided for pregnant and postpartum women and their families (69). The resource allocation for universal screening and access to information might be crucial in order to reduce the burden of disease caused by perinatal mental health disorders on the family and the society. Our study compare the impact of intervention in two different healthcare settings.

As for most randomized trials, the external validity/ generalizability of our findings could be limited sinc ewe will only target urban dwelling population who are comfortable in using internet-based applications.

For peer review only

Declarations

Ethics approval and consent to participate

The study will be conducted according to the principles of Good Clinical Practice and has been approved by the ethical review boards the Chinese University Hong Kong (IRB #2019-8170), and Shanghai Center for Women's and Children's Health (IRB #2020-F001-12). Written consent is obtained from each participant before inclusion to the study. Participation in the study is voluntary and women are free to discontinue their participation at any time and for whatever reason without explanation.

All researchers directly involved in this RCT will have training and certification in Good Clinical Practice (GCP).

For peer review only - http://bmjopen.bmj.com/site/about/guidelines.xhtml

Availability of data and material

Anonymized data will be made available to researchers on reasonable request.

Authors' contributions

SES wrote the study protocol article with the support of GA and EA. SES and GA created the study and were responsible for formulating initial study design together with HH, the local PI in Hong Kong responsible for conducting the study in Hong Kong. CHF is responsible for the recruitment of participants and set up all the epidemiological background to imitate the study in Hong Kong and Shanghai. FSC is responsible for recruitment of particiapnts. ZLP is the local PI in Shanghai, and together with DL, responsible for running the study in Shanghai. MH also helped write the protocol and conducted a literature search to inform it. All authors were involved in the development of study design and take responsibility for the final study design. SES wrote the first draft of the study protocol. All authors have contributed to the protocol development, and have read and approved the final protocol and this manuscript.

Funding

This project is funded by Swiss National Science Foundation SNSF (P2SKP3_187728). The funding agencies do not have any part in the design, data collection and analysis or reporting of this trial.

Competing interests

The authors declare that they have no competing interests.

Acknowledgements

The **Mental Health of Urban Mothers Trial (MUM-Trial)** in urban Hong Kong and Shanghai is supported by Karolinska Institutet, Chinese University of Hong Kong, and Shanghai Government`s Center for Women's and Children's Health.
We would also like to acknowledge the nonprofit organizations for their support with patient recruitment.

Trial Registration and Status

Trial registration number NCT04363177
Trial sponsor Karolinska Institute, CLINTEC, Stockholm Sweden

Figure 1 – Overview of the study and time line

For peer review only

References

1. Dayan NJ, Creveuil NC, Marks NM, Conroy NS, Herlicoviez NM, Dreyfus NM, et al. Prenatal Depression, Prenatal Anxiety, and Spontaneous Preterm Birth: A Prospective Cohort Study Among Women With Early and Regular Care. Psychosomatic Medicine. 2006;68(6):938-46.
2. Siu Bonnie W, Leung Shirley S, Ip P, Hung S, O'Hara Michael W. Antenatal risk factors for postnatal depression: a prospective study of chinese women at maternal and child health centres. BMC Psychiatry. 2012;12(1):22.
3. Eberhard-Gran M. Depression in connection with parturition. Nedstemthet og depresjon i forbindelse med födsel. 2011;40(1).
4. O'Hara MW, McCabe JE. Postpartum Depression: Current Status and Future Directions. Annual review of clinical psychology. 2013;9(1):379-407.
5. Shorey S, Chee CYI, Ng ED, Chan YH, Tam WWS, Chong YS. Prevalence and incidence of postpartum depression among healthy mothers: A systematic review and meta-analysis. Journal of psychiatric research. 2018;104:235-48.
6. Xiong R, Deng A, Wan B, Liu Y. Prevalence and factors associated with postpartum depression in women from single-child families. Int J Gynaecol Obstet. 2018;141(2):194-9.
7. Wong J, Fisher J. The role of traditional confinement practices in determining postpartum depression in women in Chinese cultures: A systematic review of the English language evidence. Journal of Affective Disorders. 2009;116(3):161-9.
8. Mu T-Y, Li Y-H, Pan H-F, Zhang L, Zha D-H, Zhang C-L, et al. Postpartum depressive mood (PDM) among Chinese women: a meta-analysis. Archives of Women's Mental Health. 2019;22(2):279-87.
9. Cameron EE, Joyce KM, Delaquis CP, Reynolds K, Protudjer JLP, Roos LE. Maternal psychological distress & mental health service use during the COVID-19 pandemic. Journal of affective disorders. 2020;276:765-74.
10. Wu Y, Zhang C, Liu H, Duan C, Li C, Fan J, et al. Perinatal depressive and anxiety symptoms of pregnant women during the coronavirus disease 2019 outbreak in China. American journal of obstetrics and gynecology. 2020;223(2):240.e1-.e9.
11. Durankuş F, Aksu E. Effects of the COVID-19 pandemic on anxiety and depressive symptoms in pregnant women: a preliminary study. The journal of maternal-fetal & neonatal medicine. 2020:1-7.
12. Brand SR, Engel SM, Canfield RL, Yehuda R. The effect of maternal PTSD following in utero trauma exposure on behavior and temperament in the 9-month-old infant. Annals of the New York Academy of Sciences. 2006;1071:454.
13. Buss C, Davis EP, Hobel CJ, Sandman CA. Maternal pregnancy-specific anxiety is associated with child executive function at 6–9 years age. Stress. 2011;14(6):665-76.
14. Stein A, Netsi E, Lawrence PJ, Granger C, Kempton C, Craske MG, et al. Mitigating the effect of persistent postnatal depression on child outcomes through an intervention to treat depression and improve parenting: a randomised controlled trial. The Lancet Psychiatry. 2018;5(2):134-44.
15. Junge C, Garthus-Niegel S, Slinning K, Polte C, Simonsen TB, Eberhard-Gran M. The Impact of Perinatal Depression on Children's Social-Emotional Development: A Longitudinal Study.(Article). Maternal and Child Health Journal. 2017;21(3):607.
16. Loh CC, Vostanis P. Perceived mother-infant relationship difficulties in postnatal depression. Infant and Child Development. 2004;13(2):159-71.

For peer review only - http://bmjopen.bmj.com/site/about/guidelines.xhtml

17. Huang L, Acharya G. Novel corona virus disease (COVID-19) in pregnancy: What clinical recommendations to follow? Acta Obstetricia et Gynecologica Scandinavica. 2020:439-42.
18. Monk C, Feng T, Lee S, Krupska I, Champagne FA, Tycko B. Distress During Pregnancy: Epigenetic Regulation of Placenta Glucocorticoid-Related Genes and Fetal Neurobehavior. American Journal of Psychiatry. 2016;173(7):705-13.
19. Doyle C, Werner E, Feng T, Lee S, Altemus M, Isler JR, et al. Pregnancy distress gets under fetal skin: Maternal ambulatory assessment & sex differences in prenatal development. Developmental Psychobiology. 2015;57(5):607-25.
20. Nelson AM. Transition to Motherhood. Oxford, UK2003. p. 465-77.
21. American Psychiatric A. Diagnostic and statistical manual of mental disorders : DSM-5. Fifth edition. ed: Arlington, VA : American Psychiatric Association; 2013.
22. R-H Xie G, He M, Walker SW, Wen SW. Postpartum Depression, Antenatal Social Support, And Postnatal Social Support. Annals of Epidemiology. 2008;18(9):736-.
23. Halbreich U, Karkun S. Cross-cultural and social diversity of prevalence of postpartum depression and depressive symptoms. Journal of Affective Disorders. 2006;91(2):97-111.
24. Glavin K, Smith L, Sørum R, Ellefsen B. Supportive counselling by public health nurses for women with postpartum depression. Journal of Advanced Nursing. 2010;66(6):1317-27.
25. Wickberg B, Bendix M, Wetterholm MB, Skalkidou A. Perinatal mental health around the world: priorities for research and service development in Sweden. BJPsych International. 2019;17(1):1-3.
26. Schwank SE, Lindgren, H., Wickberg, B., Ding, Y., Andersson, E. Perinatal Mental Health in China: Views of key health system informants in Shanghai – A Qualitative Study. Public Health Journal. 2019;3:76-81.
27. Dennis CL, Hodnett E, Kenton L, Weston J, Zupancic J, Stewart DE, et al. Effect of peer support on prevention of postnatal depression among high risk women: multisite randomised controlled trial. BMJ. 2009;338(jan15 2).
28. Solomon P. Peer Support/Peer Provided Services Underlying Processes, Benefits, and Critical Ingredients. Psychiatric Rehabilitation Journal. 2004;27(4):392-401.
29. Lau Y, Htun TP, Wong SN, Tam WSW, Klainin-Yobas P. Therapist-Supported Internet-Based Cognitive Behavior Therapy for Stress, Anxiety, and Depressive Symptoms Among Postpartum Women: A Systematic Review and Meta-Analysis. Journal of medical Internet research. 2017;19(4):e138.
30. Zhao Y, Munro-Kramer ML, Shi S, Wang J, Luo J. A randomized controlled trial: effects of a prenatal depression intervention on perinatal outcomes among Chinese high-risk pregnant women with medically defined complications. Archives of women's mental health. 2017;20(2):333.
31. Qiao YX, Wang J, Li J, Ablat A. The prevalence and related risk factors of anxiety and depression symptoms among Chinese pregnant women in Shanghai. Australian and New Zealand Journal of Obstetrics and Gynaecology. 2009;49(2):185-90.
32. Zhao Y, Kane I, Mao L, Shi S, Wang J, Lin Q, et al. The Prevalence of Antenatal Depression and its Related Factors in Chinese Pregnant Women who Present with Obstetrical Complications. Archives of Psychiatric Nursing. 2016;30(3):316-21.
33. Glavin K, Smith L, Sørum R. Prevalence of postpartum depression in two municipalities in Norway. Scandinavian Journal of Caring Sciences. 2009;23(4):705-10.
34. Koh YW, Lee AM, Chan CY, Fong DYT, Lee CP, Leung KY, et al. Survey on examining prevalence of paternal anxiety and its risk factors in perinatal period in Hong Kong: a longitudinal study. BMC public health. 2015;15(10732):1131.

For peer review only - http://bmjopen.bmj.com/site/about/guidelines.xhtml

35. Aryee S, Luk V, Leung A, Lo S. Role Stressors, Interrole Conflict, and Well-Being: The Moderating Influence of Spousal Support and Coping Behaviors among Employed Parents in Hong Kong. Journal of Vocational Behavior. 1999;54(2):259-78.
36. Kong GoH. Population. In: Department CaS, editor. Hong Kong2020.
37. Keynejad RC, Dua T, Barbui C, Thornicroft G. WHO Mental Health Gap Action Programme (mhGAP) Intervention Guide: a systematic review of evidence from low and middle-income countries. Evidence Based Mental Health. 2017;21(1).
38. Patel V. The future of psychiatry in low- and middle-income countries. Psychol Med. 2009;39(11):1759-62.
39. Webb AC, Rosso MI, Rauch LS. Internet-Based Cognitive-Behavioral Therapy for Depression: Current Progress and Future Directions. Harvard Review of Psychiatry. 2017;25(3):114-22.
40. Organization WH. Thinking healthy: a manual for psychosocial management of perinatal depression. Geneva: WHO Press; 2015.
41. Cox JL, Holden JM, Sagovsky R. Detection of postnatal depression. Development of the 10- item Edinburgh Postnatal Depression Scale. The British journal of psychiatry : the journal of mental science. 1987;150:782.
42. Lau Y, Wang Y, Yin L, Chan KS, Guo X. Validation of the Mainland Chinese version of the Edinburgh Postnatal Depression Scale in Chengdu mothers. International Journal of Nursing Studies. 2010;47(9):1139-51.
43. Dugravier R, Tubach F, Saias T, Guedeney N, Pasquet B, Purper-Ouakil D, et al. Impact of a Manualized Multifocal Perinatal Home-Visiting Program Using Psychologists on Postnatal Depression: The CAPEDP Randomized Controlled Trial.(Research Article). PLoS ONE. 2013;8(8):e72216.
44. Spitzer RL, Kroenke K, Williams JBW, Löwe B. A Brief Measure for Assessing Generalized Anxiety Disorder: The GAD-7. Archives of Internal Medicine. 2006;166(10):1092-7.
45. Tong X, An D, McGonigal A, Park S-P, Zhou D. Validation of the Generalized Anxiety Disorder-7 (GAD-7) among Chinese people with epilepsy. Epilepsy Research. 2016;120:31-6.
46. Wu K, Chan S. Psychometric Properties of the Chinese Version of the Impact of Event Scale-Revised. Hong Kong J Psychiatry. 2004;14(4):2-8.
47. Creamer M, Bell R, Failla S. Psychometric properties of the Impact of Event Scale—Revised. Behaviour Research and Therapy. 2003;41(12):1489-96.
48. Sveen J, Low A, Dyster-Aas J, Ekselius L, Willebrand M, Gerdin B. Validation of a Swedish version of the Impact of Event Scale-Revised (IES-R) in patients with burns. Journal of Anxiety Disorders. 2010;24(6):618-22.
49. Chorpita BF, Nakamura BJ. Dynamic Structure in Diagnostic Structured Interviewing: A Comparative Test of Accuracy and Efficiency. Journal of Psychopathology and Behavioral Assessment. 2008;30(1):52-60.
50. 2020 [cited 09.04.2020]. Available from: https://clincalc.com/stats/samplesize.aspx.
51. Smith-Nielsen J, Matthey S, Lange T, Vaever MS. Validation of the Edinburgh Postnatal Depression Scale against both DSM-5 and ICD-10 diagnostic criteria for depression.(Report). BMC Psychiatry. 2018;18(1).
52. Dennis C-L. Psychosocial and psychological interventions for prevention of postnatal depression: systematic review. BMJ. 2005;331(7507):15.
53. Charlson FJ, Baxter AJ, Cheng HG, Shidhaye R, Whiteford HA. The burden of mental, neurological, and substance use disorders in China and India: a systematic analysis of community representative epidemiological studies. The Lancet. 2016;388(10042):376-89.

For peer review only - http://bmjopen.bmj.com/site/about/guidelines.xhtml

54. Stein A, Pearson RM, Goodman SH, Rapa E, Rahman A, McCallum M, et al. Effects of perinatal mental disorders on the fetus and child. The Lancet. 2014;384(9956):1800-19.
55. Howard LM, Piot P, Stein A. No health without perinatal mental health. The Lancet. 2014;384(9956):1723-4.
56. Patel V, Thornicroft G. Packages of Care for Mental, Neurological, and Substance Use Disorders in Low- and Middle-Income Countries: PLoS Medicine Series. PLoS Medicine. 2009;6(10):e1000160.
57. Dennis CL, Ross LE, Grigoriadis S. Psychosocial and psychological interventions for treating antenatal depression. The Cochrane database of systematic reviews. 2007(3):CD006309.
58. Grote N, Bledsoe S. Predicting Postpartum Depressive Symptoms in New Mothers: The Role of Optimism and Stress Frequency during Pregnancy. Health & Social Work. 2007;32(2):107-18.
59. Burns A, O'Mahen H, Baxter H, Bennert K, Wiles N, Ramchandani P, et al. A pilot randomised controlled trial of cognitive behavioural therapy for antenatal depression.(Clinical report). BMC Psychiatry. 2013;13(1).
60. Austin M-P. Marcé International Society position statement on psychosocial assessment and depression screening in perinatal women. Best Practice & Research Clinical Obstetrics & Gynaecology. 2014;28(1):179-87.
61. Milgrom J, Gemmill AW. Identifying Perinatal Depression and Anxiety : Evidence-Based Practice in Screening, Psychosocial Assessment and Management. Chicester: Chicester: John Wiley & Sons, Incorporated; 2015.
62. O'donnell KJ, Bugge Jensen A, Freeman L, Khalife N, O'connor TG, Glover V. Maternal prenatal anxiety and downregulation of placental 11β-HSD2. Psychoneuroendocrinology. 2012;37(6):818-26.
63. McManus B, Nugent J. A Neurobehavioral Intervention Incorporated into a State Early Intervention Program is Associated with Higher Perceived Quality of Care Among Parents of High-Risk Newborns. Official Publication of the National Council for Community Behavioral Healthcare. 2014;41(3):381-9.
64. Dunkel Schetter C, Tanner L. Anxiety, depression and stress in pregnancy: implications for mothers, children, research, and practice. Current Opinion in Psychiatry. 2012;25(2):141-8.
65. Osma J, Barrera AZ, Ramphos E. Are Pregnant and Postpartum Women Interested in Health-Related Apps? Implications for the Prevention of Perinatal Depression. Cyberpsychology, Behavior, and Social Networking. 2016;19(6):412-5.
66. Lupton D, Pedersen S. An Australian survey of women's use of pregnancy and parenting apps. Women and Birth. 2016;29(4):368-75.
67. Schwank S, Andersson E, Fu S-C, Wickberg B, Ding Y, Lindgren H. Care-seeking behavior and disclosure on self-reported mental health among young women in urban Shanghai, China. Health Psychology Open. 2019;Accepted.
68. Schwank S, Zhou Q, He Y, Acharya G. Perinatal mental health around the world: priorities for research and service development in China. British Journal of Psychiatry International. 2020;1(1):1-4.
69. Government HK. Non-Communicable Diseases Watch: Postpartum Depression: A Public Health Problem. In: Health P, editor. 2014.

For peer review only - http://bmjopen.bmj.com/site/about/guidelines.xhtml

Figure 1 – Overview of the study and time line

Year	*Jan-March*	*April-June*	*July-September*	*October-November*	*November-December*
2020	Protocol for survey study<br><br>IRB Hong Kong, Shanghai,<br><br>Set up Survey	**T1: First peer-to-peer intervention.** Participants recruitment. **(**200 participants for each city)<br>Randomization (antenatal < week 18).<br>Web-based Peer-to-peer support at 18-22 weeks	**T2: Second peer-to-peer intervention.**<br>Web-based Peer-to-peer support at 24-28 weeks	**T3: post intervention** (34-36 weeks of gestation).<br>Psychological tests: EPDS, GAD7, IES-R, COVID-19 19 survey	**T4: post intervention** (postpartum approx. 4-6 weeks after childbirth).<br>Psychological tests: EPDS, GAD7, IES-R, COVID-19 survey
**2021**	Data collection completed	Analysis and interpretation of data	Report/manuscript writing	Reports/manuscript writing	Submission/Publication of results

For peer review only

SPIRIT

STANDARD PROTOCOL ITEMS: RECOMMENDATIONS FOR INTERVENTIONAL TRIALS

SPIRIT 2013 Checklist: Recommended items to address in a clinical trial protocol and related documents*

Section/item	Item No	Description
**Administrative information**		
Title	1	Descriptive title identifying the study design, population, interventions, and, if applicable, trial acronym **(page 1)**
Trial registration	2a	Trial identifier and registry name. If not yet registered, name of intended registry **(page 12)**
	2b	All items from the World Health Organization Trial Registration Data Set **(not applicable)**
Protocol version	3	Date and version identifier **(page 12)**
Funding	4	Sources and types of financial, material, and other support **(page 12)**
Roles and responsibilities	5a	Names, affiliations, and roles of protocol contributors **(pages 1,12)**
	5b	Name and contact information for the trial sponsor **(pages 12)**
	5c	Role of study sponsor and funders, if any, in study design; collection, management, analysis, and interpretation of data; writing of the report; and the decision to submit the report for publication, including whether they will have ultimate authority over any of these activities **(page 12)**
	5d	Composition, roles, and responsibilities of the coordinating centre, steering committee, endpoint adjudication committee, data management team, and other individuals or groups overseeing the trial, if applicable (see Item 21a for data monitoring committee) **(page 10)**
**Introduction**		
Background and rationale	6a	Description of research question and justification for undertaking the trial, including summary of relevant studies (published and unpublished) examining benefits and harms for each intervention **(pages 3,4,5)**
	6b	Explanation for choice of comparators **(not applicable)**
Objectives	7	Specific objectives or hypotheses **(page 5)**

For peer review only

Trial design	8	Description of trial design including type of trial (eg, parallel group, crossover, factorial, single group), allocation ratio, and framework (eg, superiority, equivalence, noninferiority, exploratory) **(pages 5,6)**

Methods: Participants, interventions, and outcomes

Study setting	9	Description of study settings (eg, community clinic, academic hospital) and list of countries where data will be collected. Reference to where list of study sites can be obtained **(pages 5,6)**
Eligibility criteria	10	Inclusion and exclusion criteria for participants. If applicable, eligibility criteria for study centres and individuals who will perform the interventions (eg, surgeons, psychotherapists) **(page 6)**
Interventions	11a	Interventions for each group with sufficient detail to allow replication, including how and when they will be administered **(pages 6,7)**
	11b	Criteria for discontinuing or modifying allocated interventions for a given trial participant (eg, drug dose change in response to harms, participant request, or improving/worsening disease) **(not applicable)**
	11c	Strategies to improve adherence to intervention protocols, and any procedures for monitoring adherence (eg, drug tablet return, laboratory tests) **(not applicable)**
	11d	Relevant concomitant care and interventions that are permitted or prohibited during the trial **(page 6,7)**
Outcomes	12	Primary, secondary, and other outcomes, including the specific measurement variable (eg, systolic blood pressure), analysis metric (eg, change from baseline, final value, time to event), method of aggregation (eg, median, proportion), and time point for each outcome. Explanation of the clinical relevance of chosen efficacy and harm outcomes is strongly recommended **(pages 7,8)**
Participant timeline	13	Time schedule of enrolment, interventions (including any run-ins and washouts), assessments, and visits for participants. A schematic diagram is highly recommended (see Figure) **(figure 1)**
Sample size	14	Estimated number of participants needed to achieve study objectives and how it was determined, including clinical and statistical assumptions supporting any sample size calculations **(pages 7,8)**
Recruitment	15	Strategies for achieving adequate participant enrolment to reach target sample size **(page 8)**

Methods: Assignment of interventions (for controlled trials)

Allocation:

Sequence generation	16a	Method of generating the allocation sequence (eg, computer-generated random numbers), and list of any factors for stratification. To reduce predictability of a random sequence, details of any planned restriction (eg, blocking) should be provided in a separate document that is unavailable to those who enrol participants or assign interventions **(page 6)**
Allocation concealment mechanism	16b	Mechanism of implementing the allocation sequence (eg, central telephone; sequentially numbered, opaque, sealed envelopes), describing any steps to conceal the sequence until interventions are assigned **(page 6)**
Implementation	16c	Who will generate the allocation sequence, who will enrol participants, and who will assign participants to interventions **(page 6)**
Blinding (masking)	17a	Who will be blinded after assignment to interventions (eg, trial participants, care providers, outcome assessors, data analysts), and how **(page 6)**
	17b	If blinded, circumstances under which unblinding is permissible, and procedure for revealing a participant's allocated intervention during the trial **(not applicable)**
**Methods: Data collection, management, and analysis**		
Data collection methods	18a	Plans for assessment and collection of outcome, baseline, and other trial data, including any related processes to promote data quality (eg, duplicate measurements, training of assessors) and a description of study instruments (eg, questionnaires, laboratory tests) along with their reliability and validity, if known. Reference to where data collection forms can be found, if not in the protocol **(page 7,8)**
	18b	Plans to promote participant retention and complete follow-up, including list of any outcome data to be collected for participants who discontinue or deviate from intervention protocols **(page 6)**
Data management	19	Plans for data entry, coding, security, and storage, including any related processes to promote data quality (eg, double data entry; range checks for data values). Reference to where details of data management procedures can be found, if not in the protocol **(page 8)**
Statistical methods	20a	Statistical methods for analysing primary and secondary outcomes. Reference to where other details of the statistical analysis plan can be found, if not in the protocol **(page 8)**
	20b	Methods for any additional analyses (eg, subgroup and adjusted analyses) **(page 8)**
	20c	Definition of analysis population relating to protocol non-adherence (eg, as randomised analysis), and any statistical methods to handle missing data (eg, multiple imputation) **(page 8)**

Methods: Monitoring

Data monitoring	21a	Composition of data monitoring committee (DMC); summary of its role and reporting structure; statement of whether it is independent from the sponsor and competing interests; and reference to where further details about its charter can be found, if not in the protocol. Alternatively, an explanation of why a DMC is not needed **(pages 9,10)**
	21b	Description of any interim analyses and stopping guidelines, including who will have access to these interim results and make the final decision to terminate the trial **(not applicable)**
Harms	22	Plans for collecting, assessing, reporting, and managing solicited and spontaneously reported adverse events and other unintended effects of trial interventions or trial conduct **(page 9)**
Auditing	23	Frequency and procedures for auditing trial conduct, if any, and whether the process will be independent from investigators and the sponsor **(not applicable)**

Ethics and dissemination

Research ethics approval	24	Plans for seeking research ethics committee/institutional review board (REC/IRB) approval **(page 11)**
Protocol amendments	25	Plans for communicating important protocol modifications (eg, changes to eligibility criteria, outcomes, analyses) to relevant parties (eg, investigators, REC/IRBs, trial participants, trial registries, journals, regulators) **(page 6)**
Consent or assent	26a	Who will obtain informed consent or assent from potential trial participants or authorised surrogates, and how (see Item 32) **(pages 6, 11)**
	26b	Additional consent provisions for collection and use of participant data and biological specimens in ancillary studies, if applicable
Confidentiality	27	How personal information about potential and enrolled participants will be collected, shared, and maintained in order to protect confidentiality before, during, and after the trial **(page 8)**
Declaration of interests	28	Financial and other competing interests for principal investigators for the overall trial and each study site **(page 12)**
Access to data	29	Statement of who will have access to the final trial dataset, and disclosure of contractual agreements that limit such access for investigators **(page 11)**
Ancillary and post-trial care	30	Provisions, if any, for ancillary and post-trial care, and for compensation to those who suffer harm from trial participation **(not applicable)**

For peer review only - http://bmjopen.bmj.com/site/about/guidelines.xhtml

Dissemination policy	31a	Plans for investigators and sponsor to communicate trial results to participants, healthcare professionals, the public, and other relevant groups (eg, via publication, reporting in results databases, or other data sharing arrangements), including any publication restrictions **(page 10)**
	31b	Authorship eligibility guidelines and any intended use of professional writers **(not applicable)**
	31c	Plans, if any, for granting public access to the full protocol, participant-level dataset, and statistical code **(not applicable)**
**Appendices**		
Informed consent materials	32	Model consent form and other related documentation given to participants and authorised surrogates **(not applicable)**
Biological specimens	33	Plans for collection, laboratory evaluation, and storage of biological specimens for genetic or molecular analysis in the current trial and for future use in ancillary studies, if applicable **(not applicable)**

*It is strongly recommended that this checklist be read in conjunction with the SPIRIT 2013 Explanation & Elaboration for important clarification on the items. Amendments to the protocol should be tracked and dated. The SPIRIT checklist is copyrighted by the SPIRIT Group under the Creative Commons "Attribution-NonCommercial-NoDerivs 3.0 Unported" license.

For peer review only
